# Safety and performance of surgical adhesives

**DOI:** 10.1371/journal.pone.0271531

**Published:** 2022-08-25

**Authors:** Lukasz Szymanski, Kamila Gołaszewska, Justyna Małkowska, Małgorzata Gołębiewska, Judyta Kaczyńska, Bartosz Gromadka, Damian Matak

**Affiliations:** 1 Department of Molecular Biology, Institute of Genetics and Animal Biotechnology, Polish Academy of Science, Magdalenka, Poland; 2 European Biomedical Institute, Jozefow, Poland; Saveetha Institute of Medical and Technical Sciences: Saveetha University, INDIA

## Abstract

**Background:**

Tissue adhesives are an alternative to conventional surgical sutures to reduce the time and cost of wound closure and to improve patient comfort. The use of tissue adhesives does not require any subsequent intervention and significantly lowers the volume and rate of blood loss, and reduces the need for transfusions during and after surgery. However, based on their formulation, tissue adhesives’ safety profile and functional properties may differ. Therefore, this study aimed to evaluate the basic safety and performance of NE’X Glue® Surgical Sealant, BioGlue® Surgical Sealant, and PREVELEAK^TM^ Surgical Sealant *in vitro*.

**Methods:**

The basic safety of commercially available tissue adhesives was evaluated using MEM elution assay according to ISO 10993–5 and endotoxin level according to 85. USP. The *in vitro* performance was evaluated using lap-shear by tension loading test, burst strength test, degradation, and swelling assays.

**Results:**

NE’X Glue®, BioGlue®, and PREVELEAK^TM^ did not cause cytotoxicity in MEM elution assay. All surgical adhesives are below the general limit of endotoxin contamination of 20 EU/device. NE’X Glue® and BioGlue® showed the highest and comparable strength properties in lap shear and burst strength tests compared to PREVELEAK^TM^. NE’X Glue® and PREVELEAK^TM^ are characterized by lower degradation potential than BioGlue®. PREVELEAK^TM^ is characterized by the highest swelling when compared to NE’X Glue® and BioGlue®.

**Conclusions:**

NE’X Glue® is most versatile in terms of functional properties while maintaining the same safety profile as BioGlue® and PREVELEAK^TM^.

## Introduction

Correct closure of wounds during surgical procedures as well as those sustained during injuries is an important step in applying a surgical dressing. An improperly closed wound can open up or become infected, and depending on its size and location, it can be life-threatening [1]. Surgical suturing is the most popular method of wound closure. Complications at the suture site, including infections, are one of the most common postoperative complications, which is related to the speed of the patient’s recovery and the cost of treatment, and the length of stay in the hospital [2].

Tissue adhesives have been introduced as an alternative to conventional surgical sutures to reduce the time and cost of wound closure and to improve patient comfort [3]. More than 80% of patients with sutures experience postoperative pain at the suturing site, especially during mesh fixation procedures compared to off label adhesive fixation [4–6]. The advantage of tissue adhesives is that they do not require any subsequent intervention, unlike sutures that, in some cases, need to be removed. What is more, the use of surgical adhesives significantly lowers the volume and rate of blood loss and reduces the need for transfusions during and after surgery [7–9].

Among the tissue glues, there are biological adhesives based on natural proteins (fibrin, thrombin, gelatine, albumin adhesives), synthetic glues (based on cyanoacrylate, polyethylene glycol, glutaraldehyde), biomimetic adhesives (glues secreted by lizards, mussels), and hybrid adhesives (activated by light or temperature) [10–12]. The adhesive usually consists of monomers and/or polymers functionalized with reactive groups, e.g., acrylate nitrile, thiols, and others. Their action is based on a quick, several seconds long polymerization in contact with tissue and fluids (water, blood), creating a flexible film that binds the edges of wounds [13].

Based on their formulation, the functional properties of tissue adhesives may differ. What is more, some tissue adhesives may cause adverse reactions such as tissue inflammation or necrosis. Therefore, it is crucial to determine and understand the cytotoxic potential of tissue sealants [14]. Consequently, in addition to being non-cytotoxic and biocompatible, tissue glue should have strong adhesive properties, flexibility, stability under physiological conditions, polymerize rapidly, and cause minimal swelling [15–17]. Finally, sealants should be easy to use and possibly have a hemostatic effect as well as inhibit gas leaks, e.g., air from the lungs [18]. Therefore, this study aimed to evaluate the basic safety and performance of aldehyde-albumin based formulations, NE’X Glue® Surgical Sealant, BioGlue® Surgical Sealant, and PREVELEAK^TM^ Surgical Sealant *in vitro* [7, 19].

## Results

### MEM elution

NE’X Glue® Surgical Sealant, BioGlue® Surgical Sealant, and PREVELEAK^TM^ Surgical Sealant cell culture medium extracts showed no cytotoxic potential to L-929 mouse fibroblasts using the MEM Elution method.

**Fig 1:**
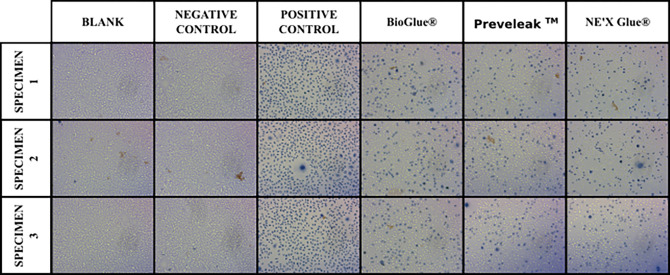
Images of cells after exposure to surgical adhesive extracts in the MEM elution study.

Results of cytotoxicity testing are presented in Table 1 and Fig 1.

**Table 1:** Results of cytotoxic potential assessment.

Sample	Grade	System Suitability
Blank	0	Valid
Negative Control	4	Valid
Positive Control	0	Valid
PREVELEAK^TM^	2	no cytotoxic potential
BioGlue®	2	no cytotoxic potential
NE’X Glue®	2	cytotoxic potential

### Determination of endotoxin level

NE’X Glue® has the lowest endotoxin contamination (0.007 EU/mL), followed by BioGlue® (0.017 EU/mL) and PREVELEAK^TM^ (0.174 EU/mL). Endotoxin testing results per 1 g finished device are presented in Table 2.

**Table 2:** Endotoxins concentration results.

Surgical Sealant	Endotoxin [E.U.] content per 1 mL of device	Endotoxin [E.U.] content per maximal size of device
NE’X Glue®	0.007	10 mL device– 0.070
BioGlue®	0.017	10 mL device– 0.170
PREVELEAK^TM^	0.174	4 mL device– 0.696

### Determination of strength properties of tissue adhesive

**Fig 2:**
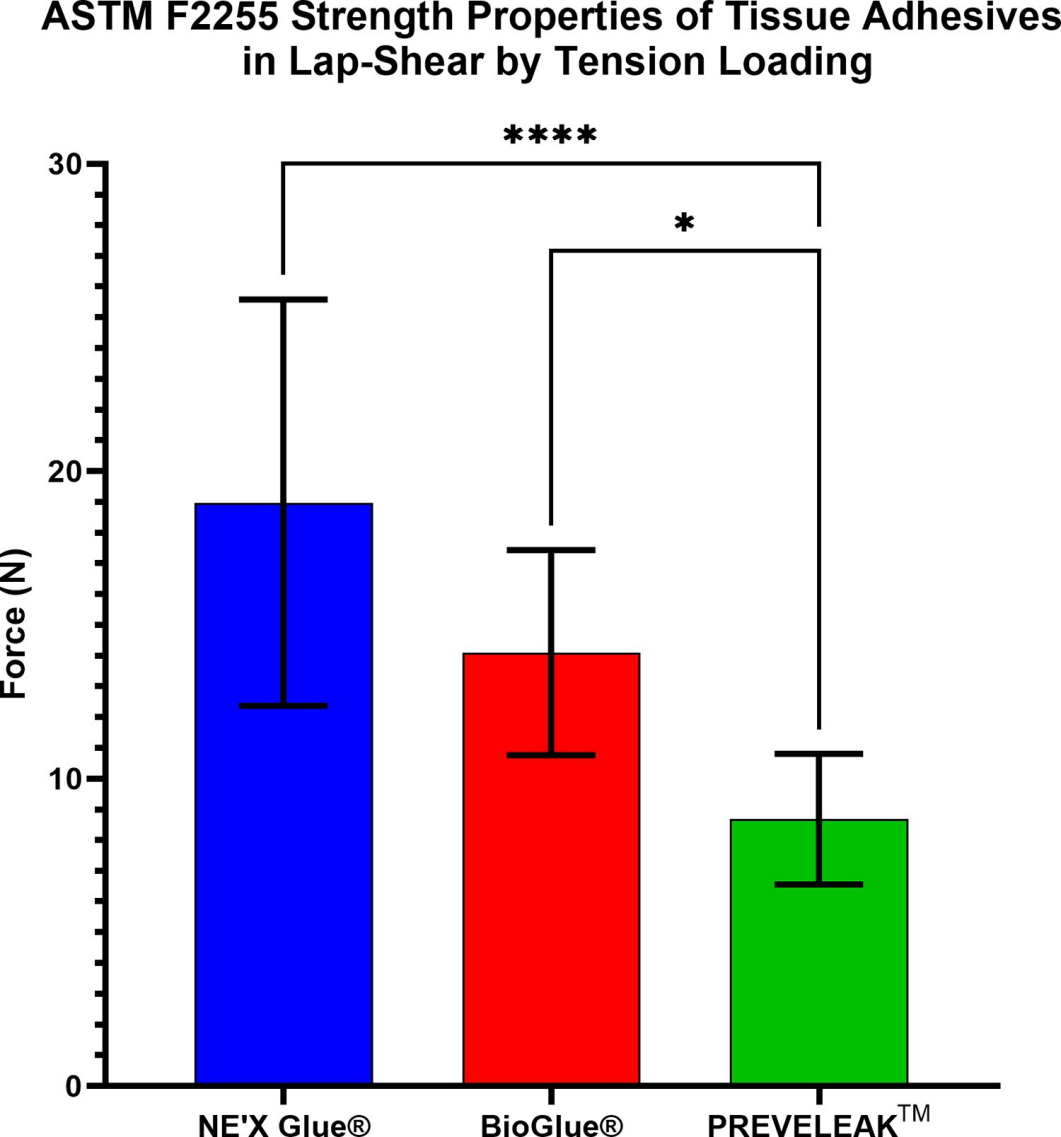
Results of ASTM F2255 strength properties of tissue adhesives in lap-shear by tension loading. n = 10 and p < 0.05 was considered statistically significant.

In the lap-shear by tension loading test according to the ASTM F2255, NE’X Glue® and BioGlue® have significantly highest strength properties (19.08 N and 14.10 N, respectively), followed by PREVELEAK^TM^ (8.11 N). Results are presented in Fig 2.

### Burst strength

**Fig 3:**
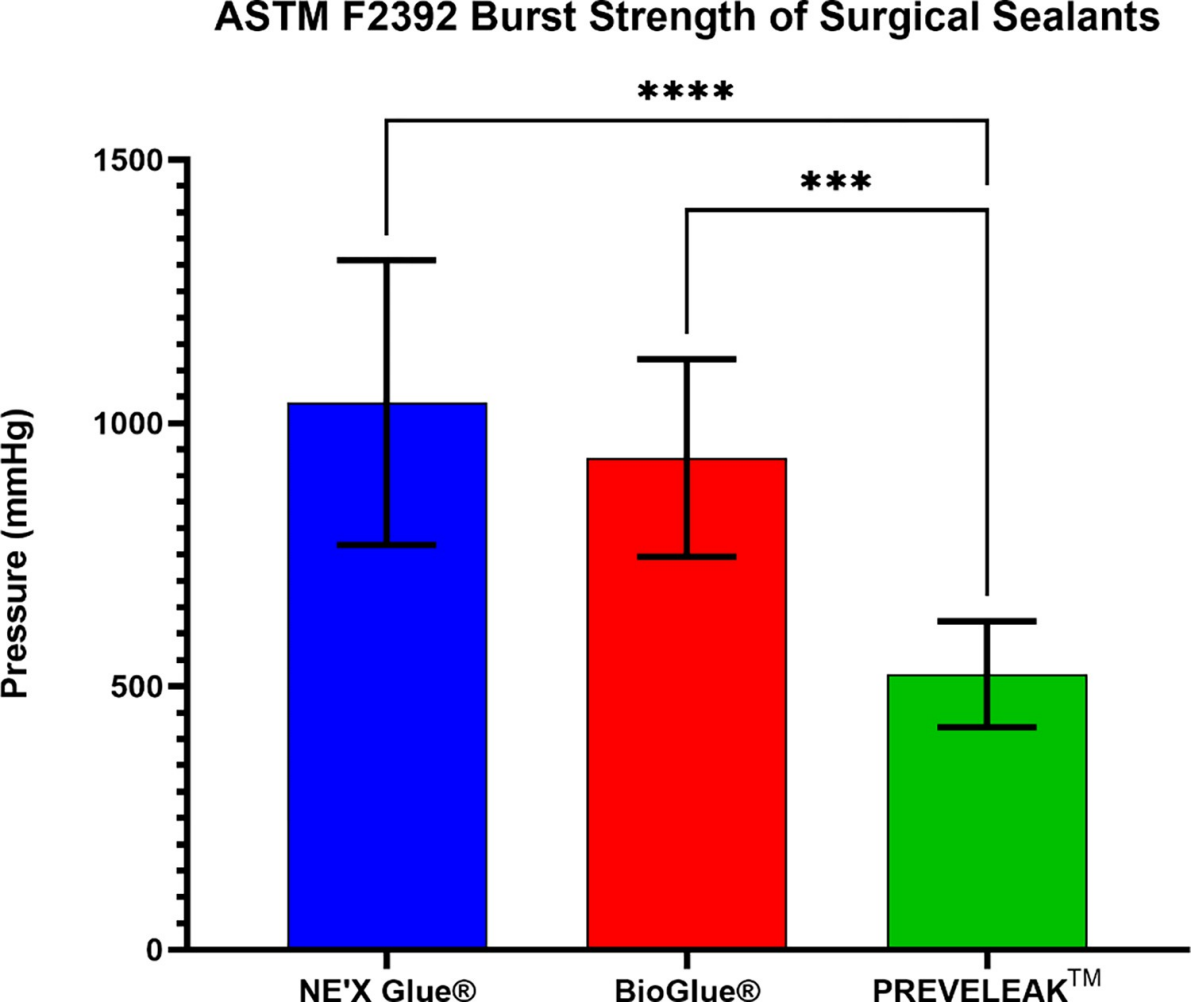
Results of ASTM F2392 burst strength of surgical sealants. n = 10 and p < 0.05 was considered statistically significant.

NE’X Glue® and BioGlue® surgical adhesives can sustain the highest vessel pressure of 1039 mmHg and 934 mmHg, respectively, while PREVELEAK^TM^ can withstand 555 mmHg. Results of ASTM F2392 Burst Strength of Surgical Sealants are presented in Fig 3.

### Degradation

**Fig 4:**
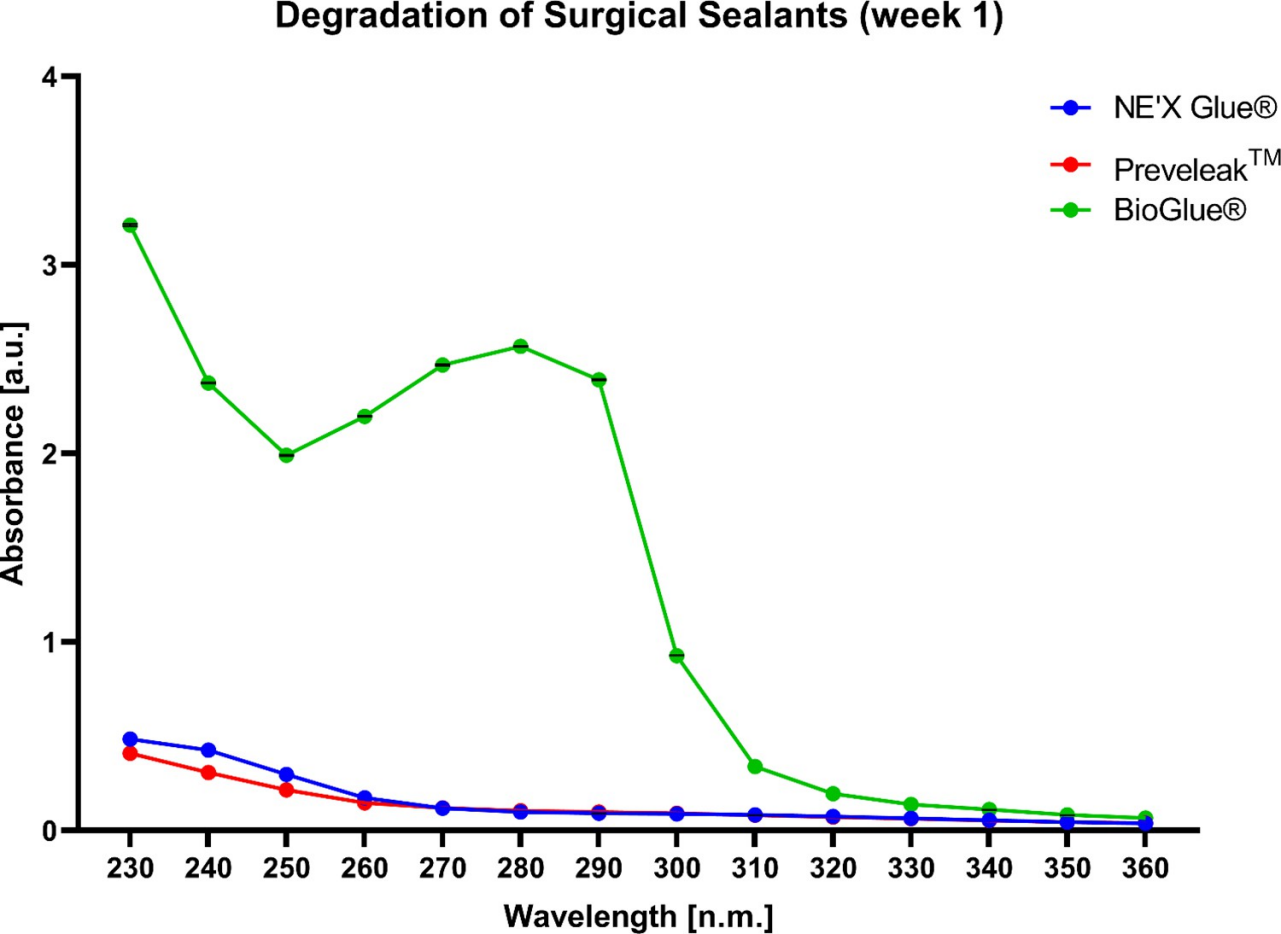
The degradation process of surgical sealants is illustrated as the absorbance spectra of water for injection after 1 week of incubation with the sample. n = 3 and p < 0.05 was considered statistically significant.

BioGlue® showed a significantly highest level of degradation after one week of incubation. NE’X Glue® and PREVELEAK^TM^ show similar levels of degradation as illustrated by the absorbance spectrum in the range of 230 to 360 nm. During the following 8 weeks, the trend has been maintained with the BioGlue® showing the highest degradation however, maximal absorbance for each sample decreased over time. Results of the degradation process after one week of incubation of surgical sealants are presented in Fig 4. The data from weeks 2–8 is presented in supplementary materials.

### Swelling

**Fig 5:**
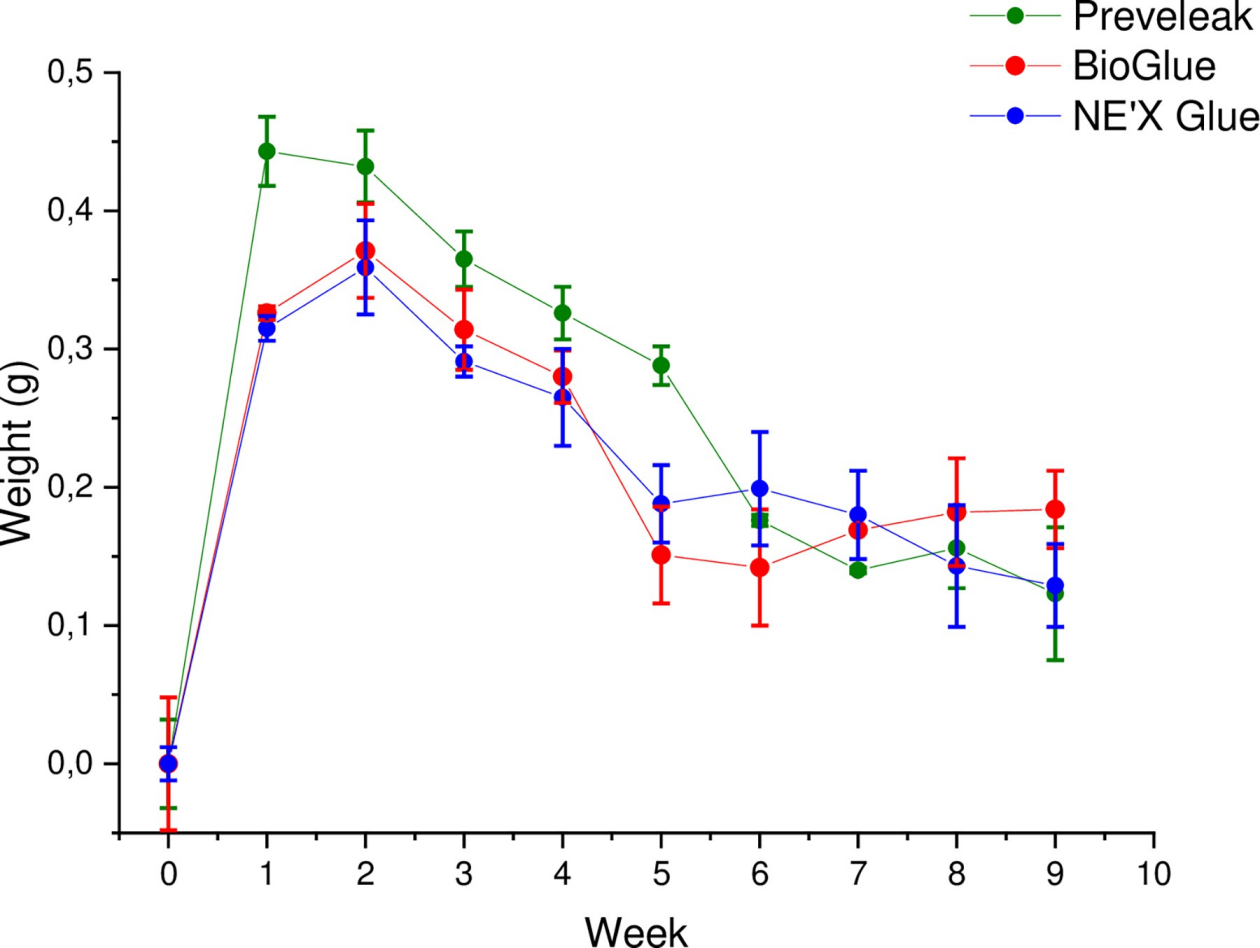
Swelling presented as a weight gain [g] over time [weeks] of surgical sealants. n = 3 and p < 0.05 was considered statistically significant.

PREVELEAK^TM^ is characterized by the highest swelling over time, especially in weeks 1–5. No differences were observed between BioGlue® and NE’X Glue® surgical adhesives. Swelling results are presented in Fig 5.

## Discussion

The aim of this study was to compare the basic safety and performance of NE’X Glue®, BioGlue®, and PREVELEAK^TM^. Safety was measured by evaluation of the cytotoxic potential and endotoxin concentration, while performance was assessed based on the lap-shear by tension loading, swelling potential, degradation potential, and burst strength.

Cytotoxicity testing allows for rapid biocompatibility assessment of the medical device. In this study, we investigated tested products in the MEM elution assays. NE’X Glue®, BioGlue®, and PREVELEAK^TM^ extracts did not cause cytotoxicity to L929 cells. Therefore, results indicate that there should be no negative consequences of using these surgical sealants in patients. In addition, tissue adhesives are commonly used directly on tissue therefore, the organs’ cell exposition is comparable in cytotoxicity tests and clinical use [20].

Endotoxin contamination in medical devices can lead to significant health complications, including acute inflammation, amyloid-beta efflux impairment, and disturbed CSF distribution. The general limit of endotoxin for medical devices intended to be used in adults is 20 EU/device. Therefore, to further test the safety of devices, endotoxin level in each tissue adhesive was evaluated. Results show that all tested tissue adhesives are well below the general limit for medical devices even for the maximal available size of the products, with NE’X Glue® having the lowest endotoxin contamination, followed by BioGlue®, and PREVELEAK^TM^. With endotoxin contamination at a low level, all tested tissue adhesives may be used in procedures involving contact with cerebrospinal fluid, for which the limit is 2.15 EU/device.

The main objective of tested adhesives often used during large blood vessel surgeries is to hold sealed tissue together. Therefore the strength properties are of crucial importance. Adhesive strength on soft tissue measured using lap-shear by tension loading test according to the ASTM F2255 showed that 2,5 cm^2^ of NE’X Glue® can withstand the force of 19.08 N, BioGlue® 14.10 N, and PREVELEAK^TM^ 8.11 N. What is more, the burst strength test showed that NE’X Glue® and BioGlue® could withstand around 1000 mmHg of pressure while PREVELEAK^TM^ can withstand around 500 mmHg of pressure. Therefore, the results indicate that all of the tested surgical adhesives can hold together large blood vessels with an average pressure of 120 mmHg as well as pathological states of 200–220 mmHg., even without additional suturing [21]. It is essential to remember, however, that internal blood pressure is not the only force influencing the artery therefore, the strength properties of NE’X Glue® and BioGlue®, which are twice as high as PREVELEAK’s^TM^, constitute a significant advantage, and the use of this tissue adhesives may be beneficial for the patient.

Tested surgical adhesives employ aldehyde solution which is mixed with protein solution during application. Over time, the degradation of cured adhesive may be a source of potentially hazardous substances that might negatively affect the patient. In this study, we showed that the degradation process of NE’X Glue® and PREVELEAK^TM^ is negligible. On the other hand, the absorbance spectra, especially in the range of 230–320 nm, of water for injection after incubation with the BioGlue® suggest a considerable degradation process. The distinctive pick in absorbance in the range 250–310 nm is characteristic for aldehydes therefore, it is possible that self-polymerization of aldehyde resulted in a portion of unreacted aldehyde that is leaching from the cured sample of BioGlue® [22, 23].

Finally, internal swelling of any kind can exert pressure on organs, surrounding tissue, or blood vessels resulting in negative consequences such as reducing the blood artery lumen. Results show that PREVELEAK^TM^ is characterized by the highest swelling over time, especially in weeks 1–5, compared to BioGlue® and NE’X Glue®. Therefore, consideration should be given to choosing the suitable surgical adhesive based on the patient’s anatomical characteristics and the operated tissue.

Overall, *in vitro* studies indicate that NE’X Glue® is the most versatile in terms of safety and functional properties among tested surgical adhesives. The differences in PREVELEAK^TM^, BioGlue®, and NE’X Glue® surgical adhesives performance is most likely attributed to the differences in the formulation, quality, and purity of reagents used for the production of the final device as well as the ratio of aldehyde to albumin [24].

## Materials and methods

Cell lines were purchased from ATCC, reagents for cell culture were purchased from Thermo Fisher Scientific, Poland, and all chemical compounds were purchased from Sigma, Poland.

Porcine skin was purchased from Stellen Medical LLC, USA.

### Statistical analysis

All results are presented as the mean ± standard deviation (SD). Statistical evaluation was performed using the two-way ANOVA with Bonferroni’s multiple comparisons test for degradation studies and one-way ANOVA with Bonferroni’s multiple comparisons test for other assays. GraphPad Prism software (version 9.3.1; GraphPad Software, Inc., La Jolla, CA, USA) was used for all evaluations. p < 0.05 was considered statistically significant. All tests were performed in triplicate unless otherwise stated. Additional statistical information is provided in supplementary materials.

### Cytotoxicity–MEM elution assay

Based on ISO 10993–12 [25], tissue adhesives were extracted in single strength MEM at 37±1°C for 72±2 hours using 0.2 g/ml extraction ratio.

Upon extraction completion, to triplicate monolayers of L929 cells, 600 μL of extracts were dosed and incubated in the presence of 5±0,1% CO2, 95% humidity for 24±1 hours. Afterward, 100 μl of freshly prepared staining solution (mixture of Trypan Blue solution with single strength MEM in 1:1 ratio) was dispended in each well. Finally, cytotoxicity was assessed by microscopic observations according to Table 1 included in ISO 10993–5 [26].

### Determination of endotoxin level

Based on ISO 10993–12 [25], an extraction ratio of 0.2 g/ml was used. Tissue adhesives were extracted in water for injection at 37±1°C for 72±2 hours.

**Fig 6:**
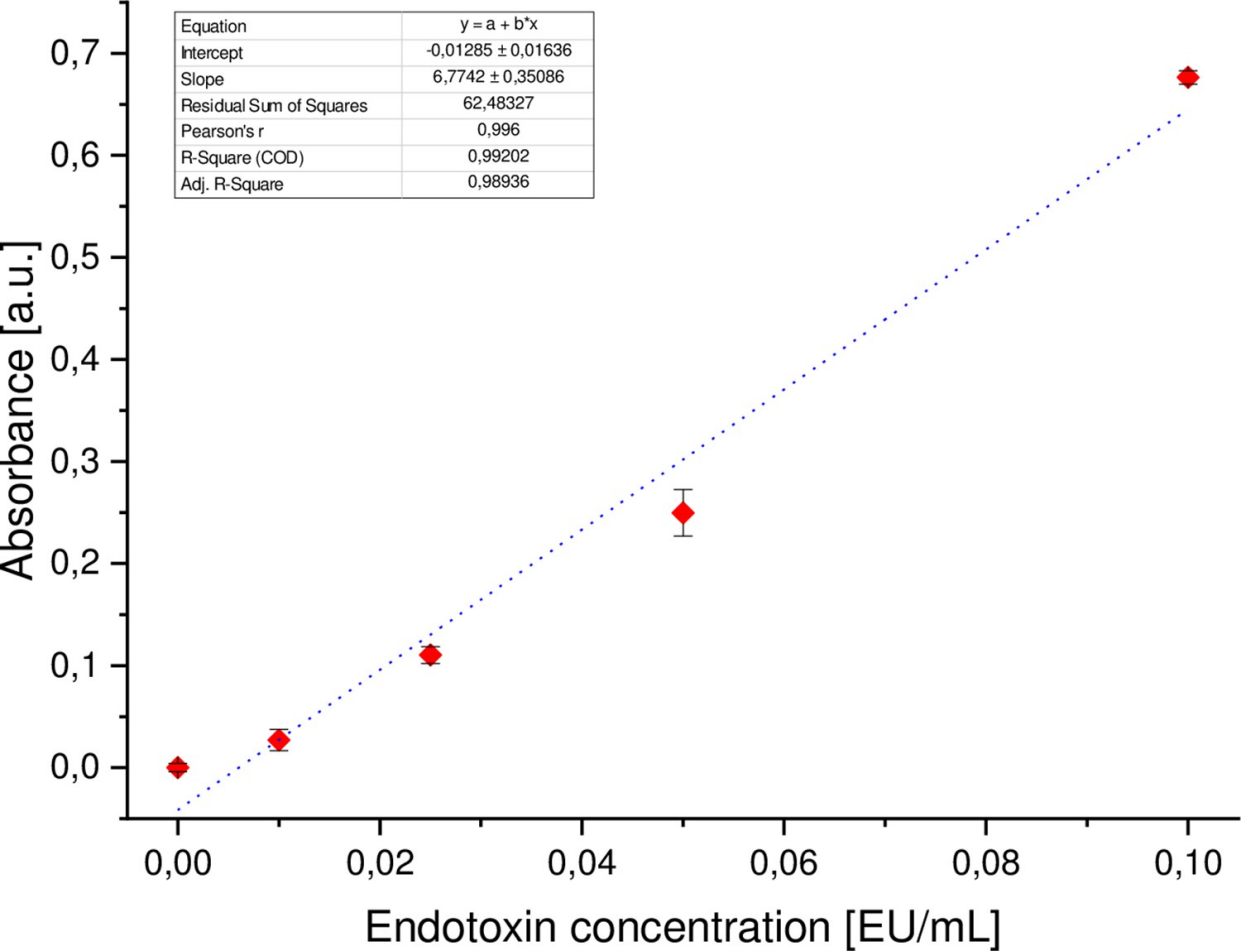
Standard curve (R^2^ = 0,992).

Pierce Chromogenic Endotoxin Quant Kit (regarding 85. Bacterial Endotoxin Test, U.S. Pharmacopoeia [22]) was used to measure endotoxin concentration. According to the manufacturer’s instruction, a standard curve (Fig 6) was prepared (R^2^ = 0,992). Internal test validation was performed by spiking the samples with a known spike of endotoxins (0,5 EU/ml). Then, after determining the respective endotoxin concentrations, the difference between the two calculated endotoxin values was calculated. The calculated value should equal the known concentration of spike ±25%.

### Determination of strength properties of tissue adhesive

**Fig 7:**
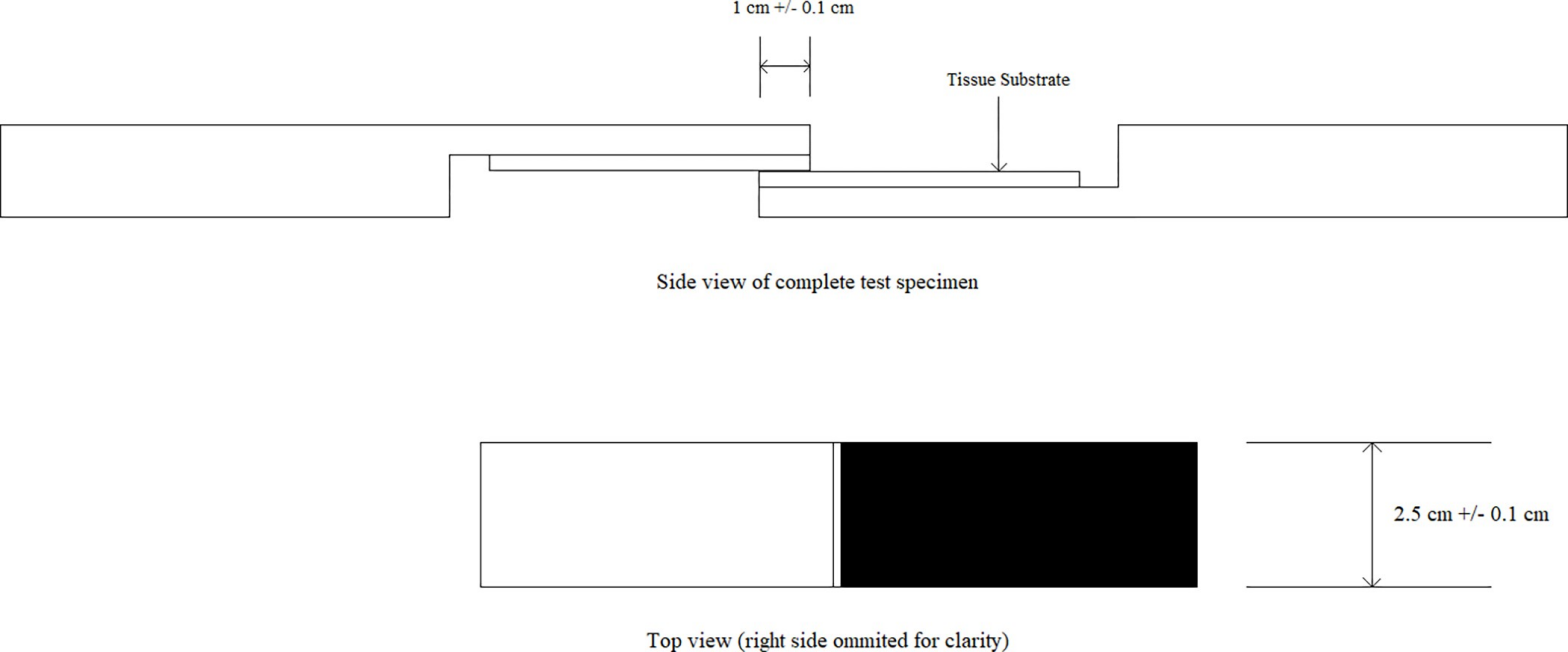
Test system scheme.

Porcine skin was cut to dimensions (Fig 7) and kept moist at all times with PBS. The backside of the tissue sample was glued to the test fixture using cyanoacrylate glue. Before testing, the test fixture was equilibrated to the temperature of 37±1°C.

Tissue adhesives were prepared according to the manufacturers’ instructions. A sufficient amount of adhesive was applied to the test fixture to coat the over-lap area uniformly without significant overflow. The two sides of the test fixture were bonded together and incubated at room temperature for 5 min.

The test specimens were wrapped with gauze soaked in PBS and were placed in an environmental chamber at 37°C for 1 hour. Before testing, the test specimens were stabilized for 15 minutes at room temperature.

**Fig 8:**
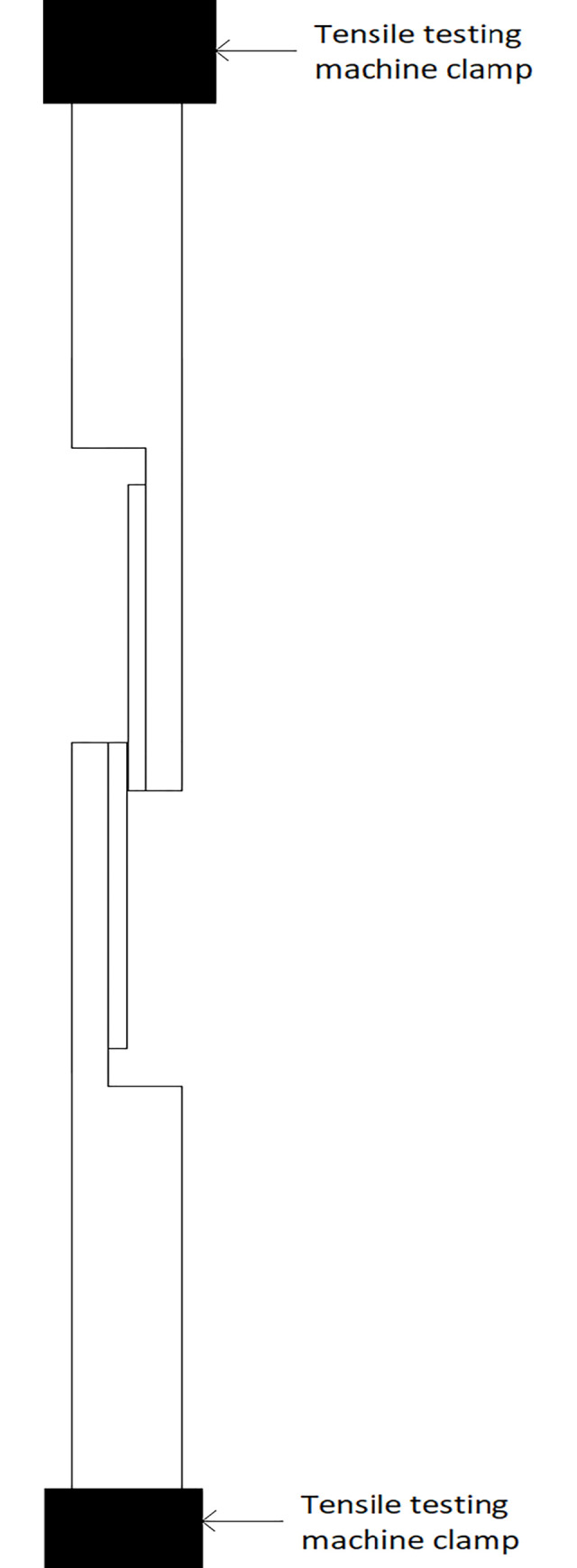
System for testing strength properties of tissue adhesive.

The test specimens were placed in tensile machine grips (Fig 8) and tested according to ASTM F2255-5 [27].

### Burst strength

**Fig 9:**
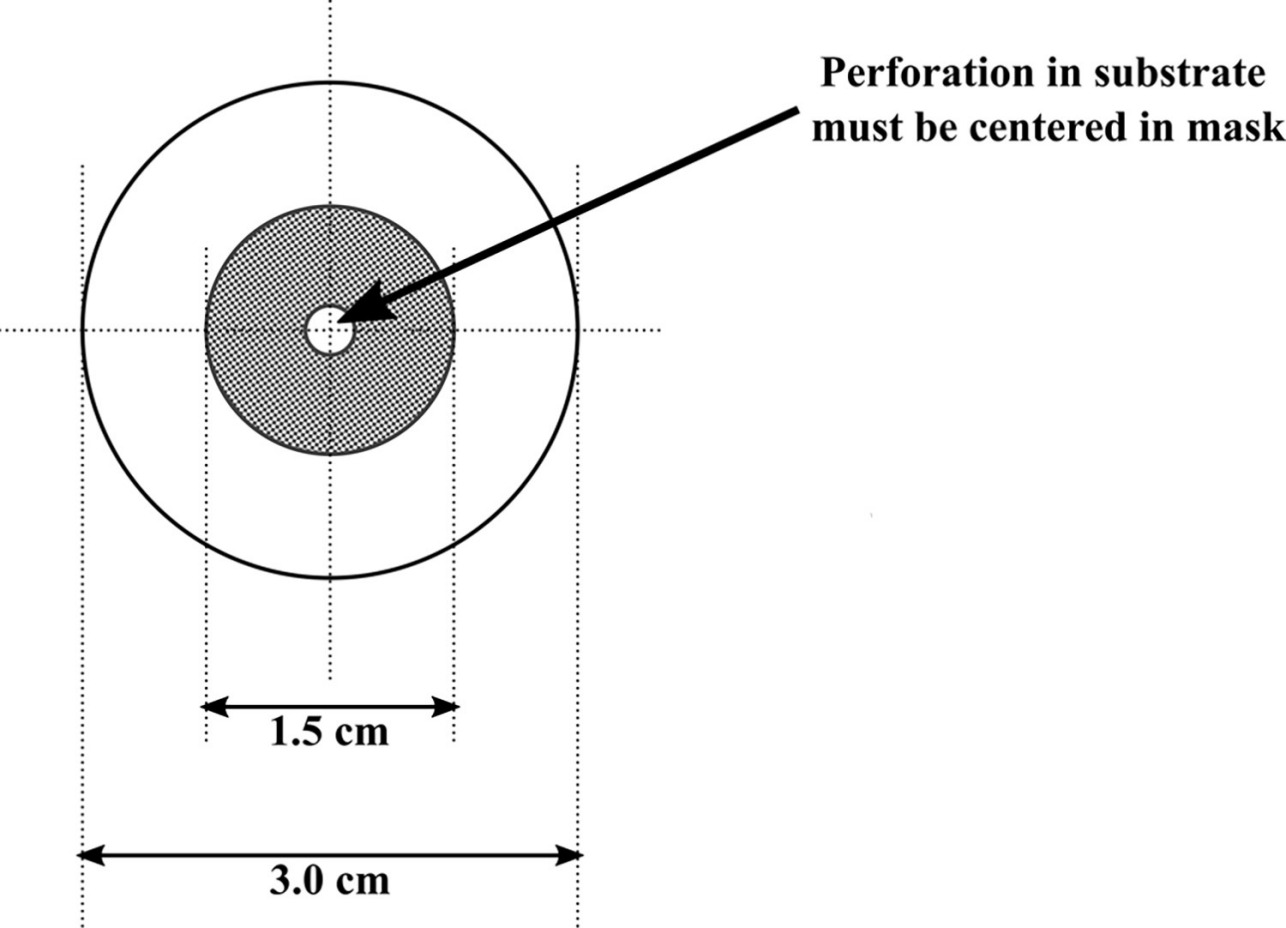
Test system scheme.

The integument was soaked with demineralized water for 5 min. Afterward, 3 cm diameter circles with holes of 3 mm diameter were cut from the integument and put on the mask with 1,5 cm diameter, as shown in Fig 9 [28].

The tested sealant was applied to the mask perforation and left to polymerize. Afterward, the probe was mounted in the apparatus. Using a syringe, water was introduced into the system. Subsequently, the apparatus was pressurized. The study was conducted until the damage causing leakage of the water occurred. The maximum pressure (in mmHg) was noted.

### Swelling

Polymerized tissue adhesive was weighted and soaked with water for injection using 0.2 g/ml ratio as indicated in ISO 10993–12 [25]. The samples were incubated at 37°C for 7 days. After that time, the water above the polymerized tissue adhesive was removed. The tissue adhesive was weighted, soaked with the same volume as on the first day of the study and incubated at 37°C for 7 days. The procedure was repeated for eight following weeks.

### Degradation

Polymerized tissue adhesive was weighted and soaked with water for injection using 0.2 g/ml ratio as indicated in ISO 10993–12 [25]. The samples were incubated at 37°C for 7 days. After that time, the water above the polymerized tissue adhesive was removed. The absorbance spectrum at 230–360 nm was measured. Then the tissue adhesive was soaked with the same volume of water for injection as on the first day of the study and incubated at 37°C for 7 days. The procedure was continued for eight following weeks.

## Supporting information

S1 File (DOCX)

## References

[1] Swathi Krishna KV, Uma Maheswari LS, Rajeswari G. Surgical glue- a promising technology for wound healing. Int J Res Pharm Sci Technol. 2018;1: 9–11. doi: 10.33974/ijrpst.v1i1.18

[2] Hranjec T, Swenson BR, Sawyer RG. Surgical Site Infection Prevention: How We Do It. Surg Infect. 2010;11: 289–294. doi: 10.1089/sur.2010.021 20518648 PMC4702440

[3] Sanders L, Nagatomi J. Clinical Applications of Surgical Adhesives and Sealants. Crit Rev Biomed Eng. 2014;42: 271–292. doi: 10.1615/critrevbiomedeng.2014011676 25597240 PMC7997729

[4] Liu H, Zheng X, Gu Y, Guo S. A Meta-Analysis Examining the Use of Fibrin Glue Mesh Fixation versus Suture Mesh Fixation in Open Inguinal Hernia Repair. Dig Surg. 2014;31: 444–451. doi: 10.1159/000370249 25592242

[5] Dąbrowiecki S, Pierściński S, Szczęsny W. The Glubran 2 glue for mesh fixation in Lichtenstein’s hernia repair: a double-blind randomized study. Videosurgery Miniinvasive Tech. 2012;2: 96–104. doi: 10.5114/wiitm.2011.27429 23256009 PMC3516972

[6] Apfelbaum JL, Chen C, Mehta SS, Gan TJ. Postoperative pain experience: results from a national survey suggest postoperative pain continues to be undermanaged. Anesth Analg. 2003;97: 534–540. doi: 10.1213/01.ANE.0000068822.10113.9E 12873949

[7] Bahouth Z, Moskovitz B, Halachmi S, Nativ O. Bovine serum albumin–glutaraldehyde (BioGlue®) tissue adhesive versus standard renorrhaphy following renal mass enucleation: a retrospective comparison. Ther Adv Urol. 2017;9: 67–72. doi: 10.1177/1756287217697662 28392835 PMC5378098

[8] Hidas G, Kastin A, Mullerad M, Shental J, Moskovitz B, Nativ O. Sutureless nephron-sparing surgery: Use of albumin glutaraldehyde tissue adhesive (BioGlue). Urology. 2006;67: 697–700. doi: 10.1016/j.urology.2005.10.064 16566976

[9] Hewitt CW, Marra SW, Kann BR, Tran HS, Puc MM, Chrzanowski FA, et al. BioGlue surgical adhesive for thoracic aortic repair during coagulopathy: efficacy and histopathology. Ann Thorac Surg. 2001;71: 1609–1612. doi: 10.1016/s0003-4975(01)02424-9 11383808

[10] Ayyildiz SN, Ayyildiz A. Cyanoacrylic tissue glues: Biochemical properties and their usage in urology. Türk Ürol DergisiTurkish J Urol. 2017;43: 14–24. doi: 10.5152/tud.2017.09465 28270946 PMC5330263

[11] Bochyńska AI, Hannink G, Grijpma DW, Buma P. Tissue adhesives for meniscus tear repair: an overview of current advances and prospects for future clinical solutions. J Mater Sci Mater Med. 2016;27: 85. doi: 10.1007/s10856-016-5694-5 26970767 PMC4789195

[12] Fortelny RH, Petter-Puchner AH, Glaser KS, Redl H. Use of fibrin sealant (Tisseel/Tissucol) in hernia repair: a systematic review. Surg Endosc. 2012;26: 1803–1812. doi: 10.1007/s00464-012-2156-0 22278103

[13] Sinha S. A SINGLE BLIND, PROSPECTIVE, RANDOMIZED TRIAL COMPARING N-BUTYL 2-CYANOACRYLATE TISSUE ADHESIVE (INDERMIL) AND SUTURES FOR SKIN CLOSURE IN HAND SURGERY. J Hand Surg J Br Soc Surg Hand. 2001;26: 264–265. doi: 10.1054/jhsb.2000.0572 11386782

[14] Wang Y, Liu W, Yang X. Systemic contact dermatitis caused by a surgical glue. Adv Dermatol Allergol. 2021;38: 338–339. doi: 10.5114/ada.2021.106215 34408601 PMC8362753

[15] Perrin BRM, Dupeux M, Tozzi P, Delay D, Gersbach P, von Segesser LK. Surgical glues: are they really adhesive? Eur J Cardiothorac Surg. 2009;36: 967–972. doi: 10.1016/j.ejcts.2009.06.026 19643616

[16] Donkerwolcke M, Burny F, Muster D. Tissues and bone adhesives—historical aspects. Biomaterials. 1998;19: 1461–1466. doi: 10.1016/s0142-9612(98)00059-3 9794519

[17] Bhagat V, Becker ML. Degradable Adhesives for Surgery and Tissue Engineering. Biomacromolecules. 2017;18: 3009–3039. doi: 10.1021/acs.biomac.7b00969 28862846

[18] Araki M, Tao H, Nakajima N, Sugai H, Sato T, Hyon S-H, et al. Development of new biodegradable hydrogel glue for preventing alveolar air leakage. J Thorac Cardiovasc Surg. 2007;134: 1241–1248. doi: 10.1016/j.jtcvs.2007.07.020 17976456

[19] Florek H-J, Brunkwall J, Orend K-H, Handley I, Pribble J, Dieck R. Results from a First-in-Human Trial of a Novel Vascular Sealant. Front Surg. 2015;2: 29. doi: 10.3389/fsurg.2015.00029 26191528 PMC4486749

[20] Liu X, Rodeheaver DP, White JC, Wright AM, Walker LM, Zhang F, et al. A comparison of in vitro cytotoxicity assays in medical device regulatory studies. Regul Toxicol Pharmacol RTP. 2018;97: 24–32. doi: 10.1016/j.yrtph.2018.06.003 29885342

[21] Physiology, Cardiac Cycle—StatPearls—NCBI Bookshelf. [cited 3 Mar 2022]. Available: https://www.ncbi.nlm.nih.gov/books/NBK459327/?fbclid=IwAR1HNj45d4_xk2VYlDlyoBzFtjytdWjIv-sWzunMpO-B_wl43Nsc9gI_uvE

[22] Migneault I, Dartiguenave C, Bertrand MJ, Waldron KC. Glutaraldehyde: behavior in aqueous solution, reaction with proteins, and application to enzyme crosslinking. BioTechniques. 2004;37: 790–796, 798–802. doi: 10.2144/04375RV01 15560135

[23] Mot AC, Roman A, Lupan I, Kurtz DM, Silaghi-Dumitrescu R. Towards the Development of Hemerythrin-Based Blood Substitutes. Protein J. 2010;29: 387–393. doi: 10.1007/s10930-010-9264-2 20582620

[24] Bao Z, Gao M, Sun Y, Nian R, Xian M. The recent progress of tissue adhesives in design strategies, adhesive mechanism and applications. Mater Sci Eng C Mater Biol Appl. 2020;111: 110796. doi: 10.1016/j.msec.2020.110796 32279807

[25] ISO 10993–12:2021(en), Biological evaluation of medical devices—Part 12: Sample preparation and reference materials. [cited 13 Jan 2022]. Available: https://www.iso.org/obp/ui/#iso:std:iso:10993:-12:ed-5:v1:en

[26] ISO 10993–5:2009(en), Biological evaluation of medical devices —Part 5: Tests for in vitro cytotoxicity. [cited 13 Jan 2022]. Available: https://www.iso.org/obp/ui/#iso:std:iso:10993:-5:ed-3:v1:en

[27] F04 Committee. Test Method for Strength Properties of Tissue Adhesives in Lap-Shear by Tension Loading. ASTM International; doi: 10.1520/F2255-05R15

[28] F04 Committee. Test Method for Burst Strength of Surgical Sealants. ASTM International; doi: 10.1520/F2392-04R15

