# Correction: Safety and performance of surgical adhesives

**DOI:** 10.1371/journal.pone.0294614

**Published:** 2023-11-15

**Authors:** Lukasz Szymanski, Kamila Gołaszewska, Justyna Małkowska, Małgorzata Gołębiewska, Judyta Kaczyńska, Bartosz Gromadka, Damian Matak

There is an error in Table 1. The "System Suitability" for "NE’X Glue®" should be "no cytotoxic potential".

**Table 1:** Results of cytotoxic potential assessment.

Sample	Grade	System Suitability
Blank	0	Valid
Negative Control	4	Valid
Positive Control	0	Valid
PREVELEAK^TM^	2	no cytotoxic potential
BioGlue®	2	no cytotoxic potential
NE’X Glue®	2	no cytotoxic potential
