# Supplementary material for: Safety and performance of surgical adhesives
Source: PLoS One. 2022 Aug 25;17(8):e0271531. doi: 10.1371/journal.pone.0271531 (PMC9409600; doi:10.1371/journal.pone.0271531)
Supplement: S1 File — (DOCX) [file pone.0271531.s001.docx]

***RESULTS***

**Additional information for pressure on the vessel**

Table S1. Results of ASTM F2392 Burst Strength of Surgical Sealants

| **Surgical Sealant** | **Pressure  [mmHg]** | **Standard Deviation [mmHg]** |
| --- | --- | --- |
| NE’X Glue® | 1038.82 | 270.67 |
| BioGlue® | 933.90 | 187.60 |
| PREVELEAK | 523.36 | 100.63 |

Table S2. Result of one-way ANOVA with Bonferroni's multiple comparisons test for pressure on the vessel

| **Bonferroni's multiple comparisons test** | **Mean Diff.** | **Summary** | **Adjusted P Value** |
| --- | --- | --- | --- |
| NE’X Glue vs. BioGlue | 104.9 | ns | 0.71733 |
| NE’X Glue vs. PREVELEAK | 515.5 | **** | <0.0001 |
| BioGlue vs. PREVELEAK | 410.5 | *** | 0.0002 |

**
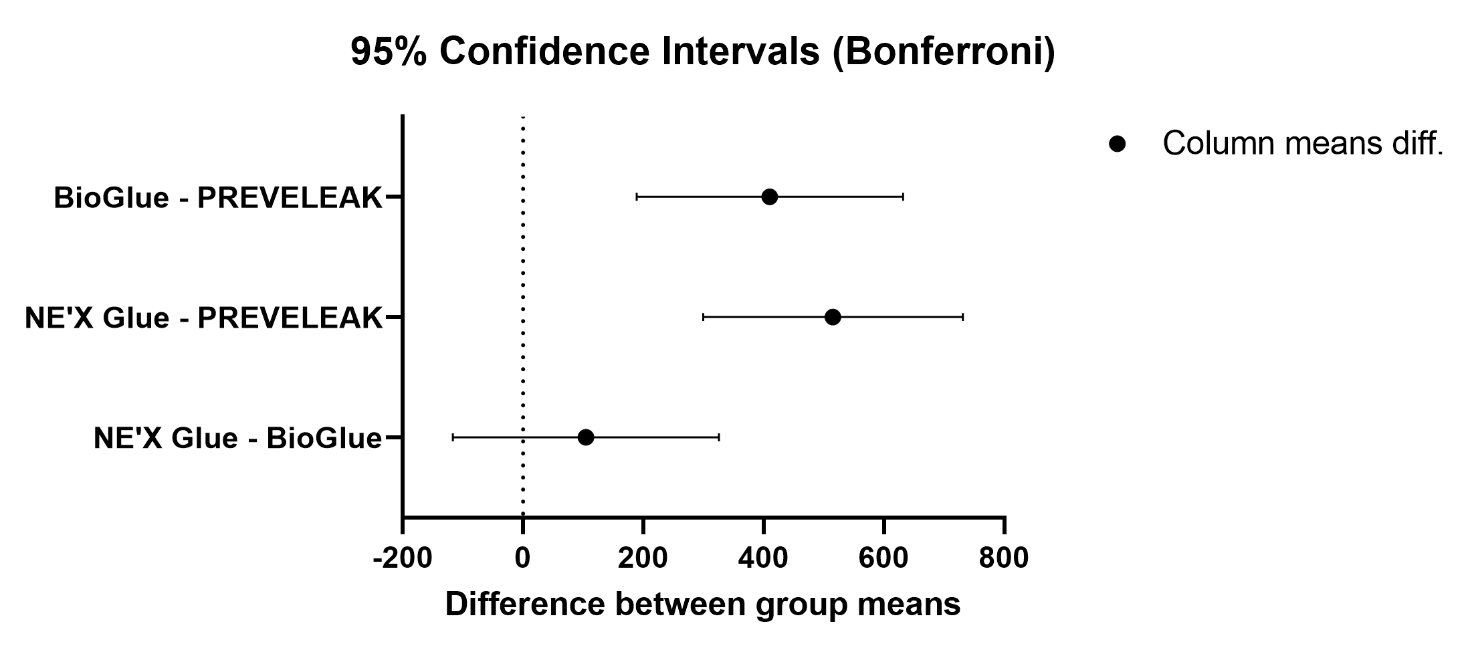
**

**Additional information for swelling**

Table S3. Results of swelling of surgical sealants

|  | **Weight [g]** | | | | | | | | | |
| --- | --- | --- | --- | --- | --- | --- | --- | --- | --- | --- |
| Weeks ► | 0 | 1 | 2 | 3 | 4 | 5 | 6 | 7 | 8 | 9 |
| PREVELEAK | 16.82 | 17.263 | 17.252 | 17.185 | 17.146 | 17.108 | 16.996 | 16.96 | 16.976 | 16.943 |
| BioGlue® | 17.823 | 18.149 | 18.194 | 18.137 | 18.103 | 17.974 | 17.965 | 17.992 | 18.005 | 18.007 |
| NE’X Glue® | 17.653 | 17.968 | 18.012 | 17.944 | 17.918 | 17.841 | 17.852 | 17.833 | 17.796 | 17.782 |

**Additional information for determination of strength properties of tissue adhesive**

Table S4. Results of ASTM F2255 Strength Properties of Tissue Adhesives in Lap-Shear by Tension Loading

| **Surgical Sealant** | **Force [N]** | **Standard Deviation [N]** |
| --- | --- | --- |
| NE’X Glue® | 19.08 | 6.53 |
| BioGlue® | 14.10 | 3.34 |
| PREVELEAK | 8.58 | 1.88 |

Table S5. Result of one-way ANOVA with Bonferroni's multiple comparisons test for determination of strength properties of tissue adhesive

| **Bonferroni's multiple comparisons test** | **Mean Diff.** | **Summary** | **Adjusted P Value** |
| --- | --- | --- | --- |
| NE’X Glue vs. BioGlue | 4.876 | ns | 0.0629 |
| NE’X Glue vs. PREVELEAK | 10.29 | **** | <0.0001 |
| BioGlue vs. PREVELEAK | 5.417 | * | 0.0335 |


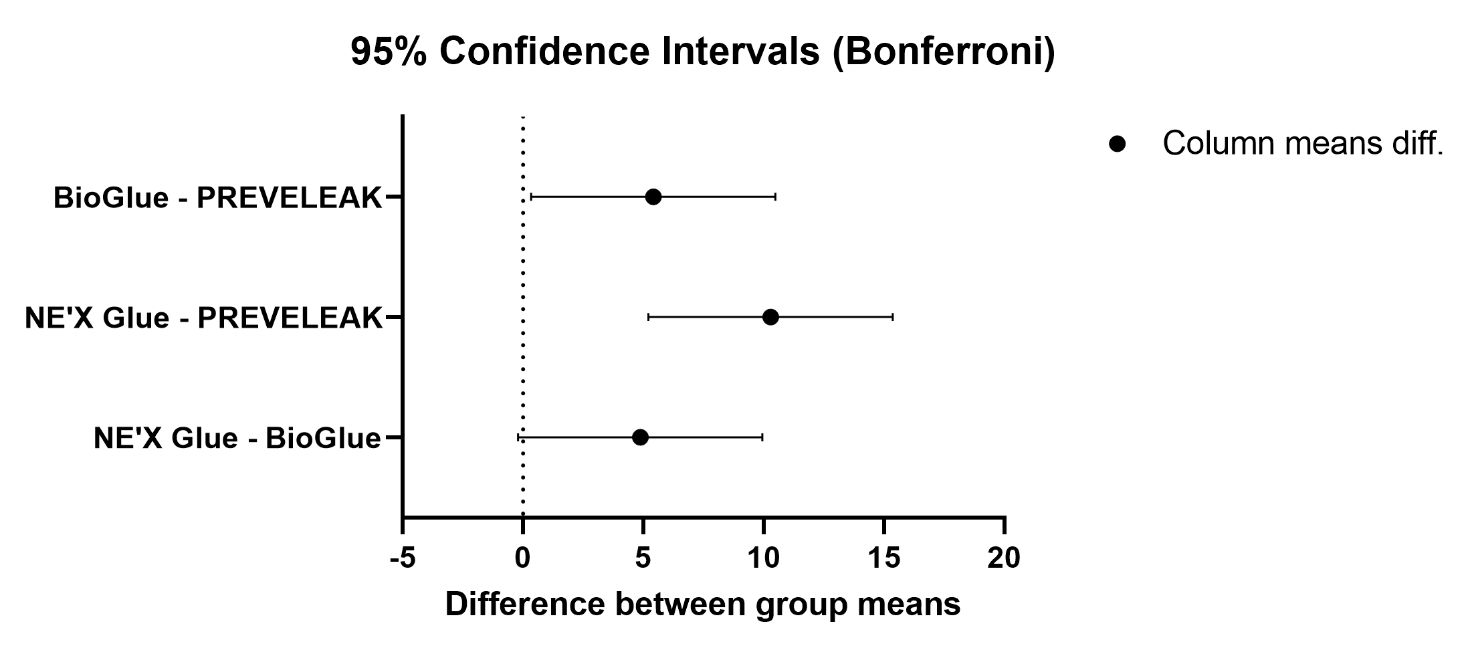


**Additional information for swelling**

Figure S6. Images of Surgical Sealants swelling process.


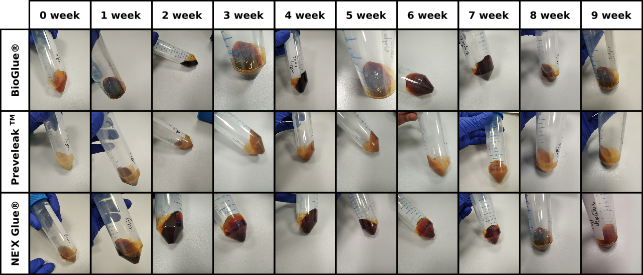


**Additional information for degradation of surgical sealants**

Figure S7. Degradation process of BioGlue

Figure S8. Degradation process of PREVELEAK

Figure S9. Degradation process of NE’X Glue

Table S10. Degradation process of surgical sealants

| **Week 1** | **Week 2** | **Week 3** |
| --- | --- | --- |
| 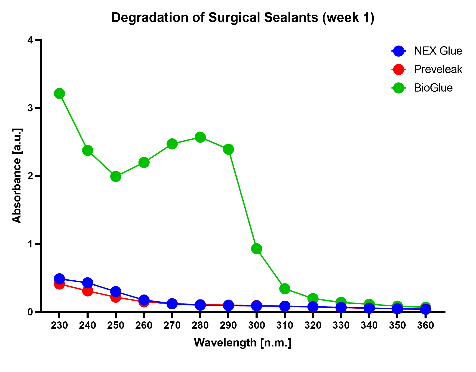 | 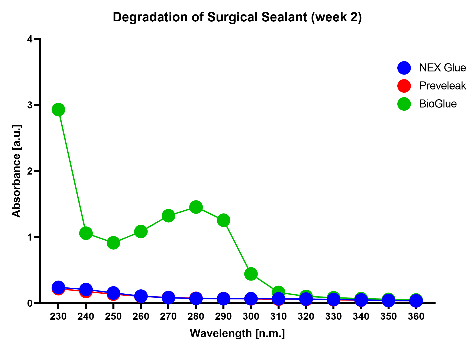 | 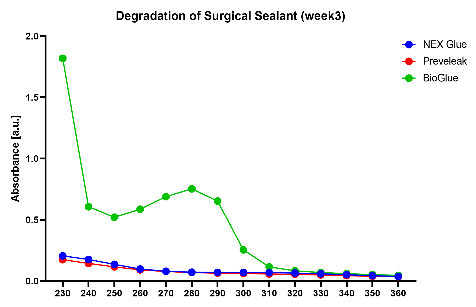 |
| **Week 4** | **Week 5** | **Week 6** |
| 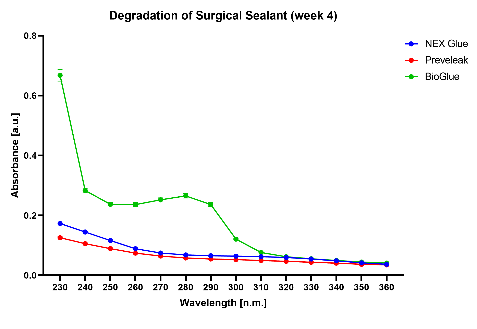 | 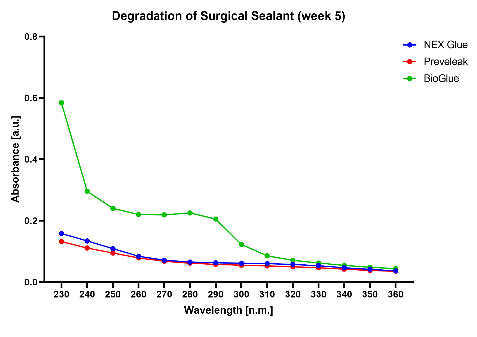 | 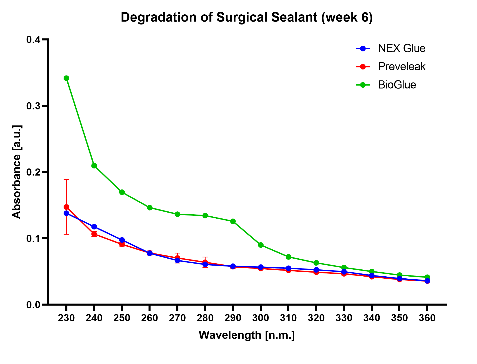 |
| **Week 7** | **Week 8** | **Week 9** |
| 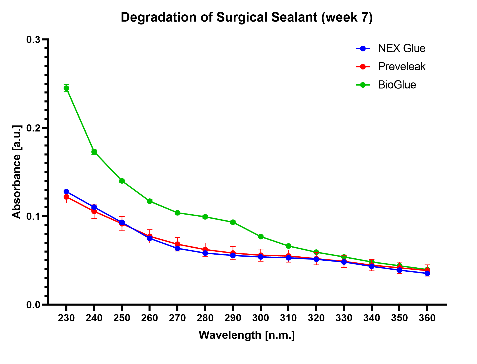 | 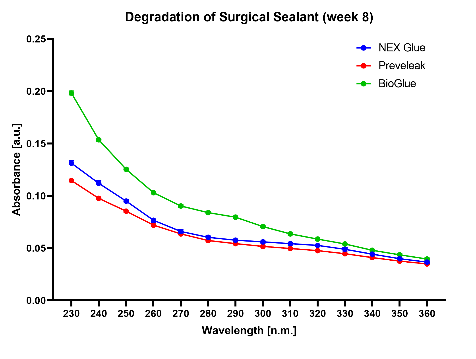 | 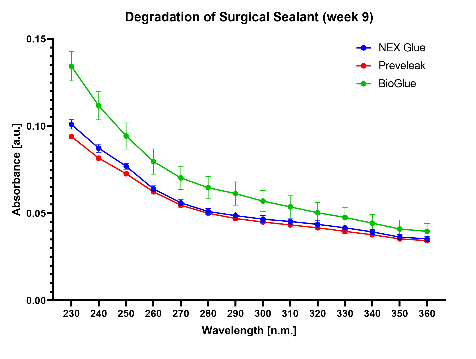 |

Table S11. Result of two-way ANOVA with Bonferroni's multiple comparisons test for degradation of surgical sealants for week 1

| **Week** | **Wavelength**  **[nm]** | **Bonferroni's multiple comparisons test** | **Mean Diff.** | **Summary** | **Adjusted P Value** |
| --- | --- | --- | --- | --- | --- |
| 1 | 230 | NEX Glue vs. Preveleak | 0,07600 | **** | <0,0001 |
|  |  | NEX Glue vs. BioGlue | -2,727 | **** | <0,0001 |
|  |  | Preveleak vs. BioGlue | -2,803 | **** | <0,0001 |
|  | 240 | NEX Glue vs. Preveleak | 0,1200 | **** | <0,0001 |
|  |  | NEX Glue vs. BioGlue | -1,947 | **** | <0,0001 |
|  |  | Preveleak vs. BioGlue | -2,067 | **** | <0,0001 |
|  | 250 | NEX Glue vs. Preveleak | 0,08133 | **** | <0,0001 |
|  |  | NEX Glue vs. BioGlue | -1,693 | **** | <0,0001 |
|  |  | Preveleak vs. BioGlue | -1,774 | **** | <0,0001 |
|  | 260 | NEX Glue vs. Preveleak | 0,02700 | **** | <0,0001 |
|  |  | NEX Glue vs. BioGlue | -2,024 | **** | <0,0001 |
|  |  | Preveleak vs. BioGlue | -2,051 | **** | <0,0001 |
|  | 270 | NEX Glue vs. Preveleak | -0,001000 | **** | <0,0001 |
|  |  | NEX Glue vs. BioGlue | -2,352 | **** | <0,0001 |
|  |  | Preveleak vs. BioGlue | -2,351 | **** | <0,0001 |
|  | 280 | NEX Glue vs. Preveleak | -0,007000 | **** | <0,0001 |
|  |  | NEX Glue vs. BioGlue | -2,471 | **** | <0,0001 |
|  |  | Preveleak vs. BioGlue | -2,464 | **** | <0,0001 |
|  | 290 | NEX Glue vs. Preveleak | -0,007333 | ** | 0,0062 |
|  |  | NEX Glue vs. BioGlue | -2,300 | **** | <0,0001 |
|  |  | Preveleak vs. BioGlue | -2,293 | **** | <0,0001 |
|  | 300 | NEX Glue vs. Preveleak | -0,002667 | * | 0,0458 |
|  |  | NEX Glue vs. BioGlue | -0,8397 | **** | <0,0001 |
|  |  | Preveleak vs. BioGlue | -0,8370 | **** | <0,0001 |
|  | 310 | NEX Glue vs. Preveleak | 0,001333 | ns | 0,1716 |
|  |  | NEX Glue vs. BioGlue | -0,2577 | **** | <0,0001 |
|  |  | Preveleak vs. BioGlue | -0,2590 | **** | <0,0001 |
|  | 320 | NEX Glue vs. Preveleak | 0,002000 | **** | <0,0001 |
|  |  | NEX Glue vs. BioGlue | -0,1210 | **** | <0,0001 |
|  |  | Preveleak vs. BioGlue | -0,1230 | **** | <0,0001 |
|  | 330 | NEX Glue vs. Preveleak | 0,002000 | * | 0,0397 |
|  |  | NEX Glue vs. BioGlue | -0,07433 | **** | <0,0001 |
|  |  | Preveleak vs. BioGlue | -0,07633 | **** | <0,0001 |
|  | 340 | NEX Glue vs. Preveleak | 0,002000 | **** | <0,0001 |
|  |  | NEX Glue vs. BioGlue | -0,05567 | *** | 0,0001 |
|  |  | Preveleak vs. BioGlue | -0,05767 | *** | 0,0001 |
|  | 350 | NEX Glue vs. Preveleak | -0,0003333 | ns | >0,9999 |
|  |  | NEX Glue vs. BioGlue | -0,03800 | **** | <0,0001 |
|  |  | Preveleak vs. BioGlue | -0,03767 | **** | <0,0001 |
|  | 360 | NEX Glue vs. Preveleak | -0,001000 | **** | <0,0001 |
|  |  | NEX Glue vs. BioGlue | -0,02800 | **** | <0,0001 |
|  |  | Preveleak vs. BioGlue | -0,02700 | **** | <0,0001 |


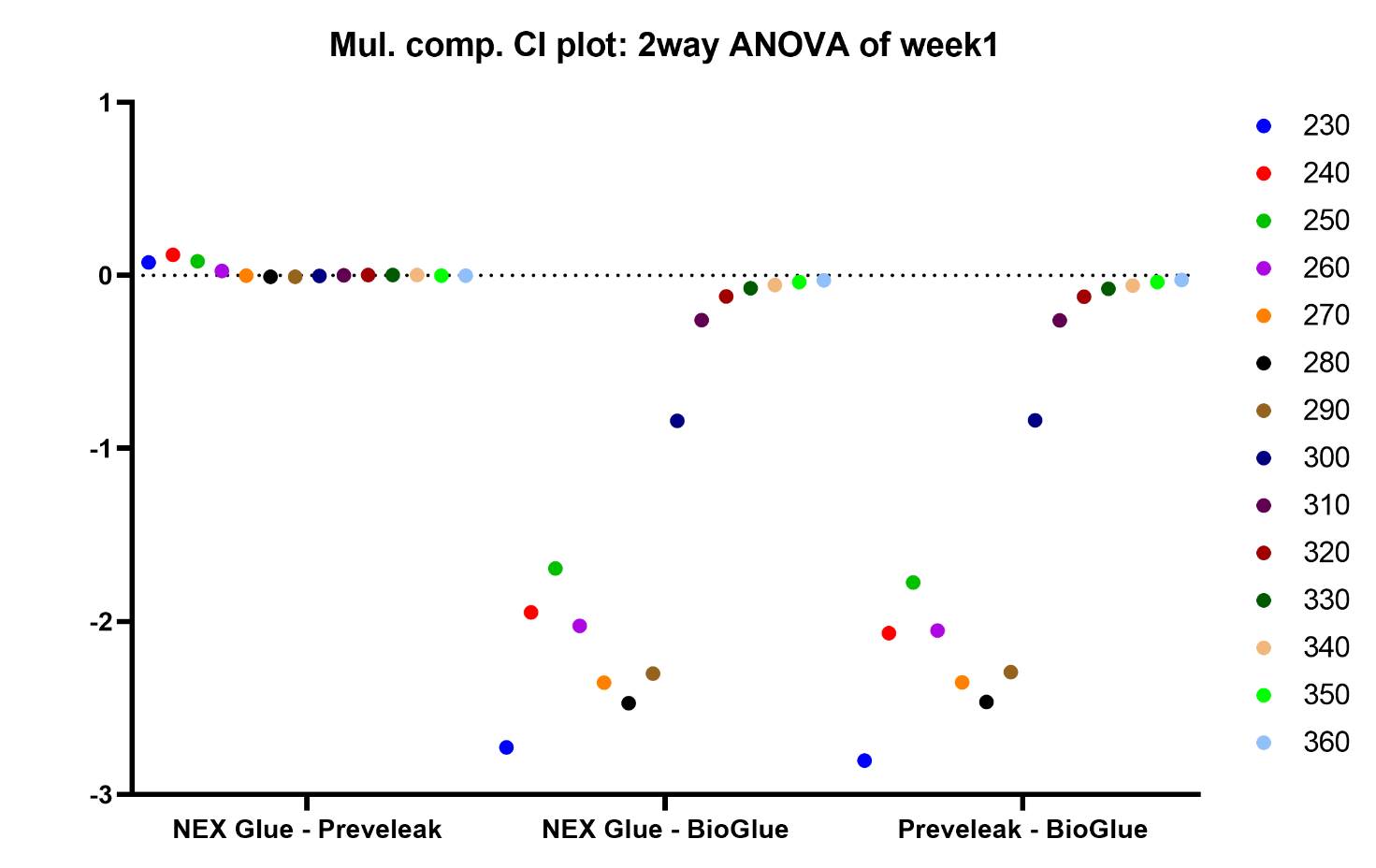


Table S12. Result of two-way ANOVA with Bonferroni's multiple comparisons test for degradation of surgical sealants for week 2

| **Week** | **Wavelength**  **[nm]** | **Bonferroni's multiple comparisons test** | **Mean Diff.** | **Summary** | **Adjusted P Value** |
| --- | --- | --- | --- | --- | --- |
| 2 | 230 | NEX Glue vs. Preveleak | 0,01967 | *** | 0,0004 |
|  |  | NEX Glue vs. BioGlue | -2,691 | **** | <0,0001 |
|  |  | Preveleak vs. BioGlue | -2,711 | **** | <0,0001 |
|  | 240 | NEX Glue vs. Preveleak | 0,03133 | **** | <0,0001 |
|  |  | NEX Glue vs. BioGlue | -0,8520 | **** | <0,0001 |
|  |  | Preveleak vs. BioGlue | -0,8833 | **** | <0,0001 |
|  | 250 | NEX Glue vs. Preveleak | 0,01933 | *** | 0,0009 |
|  |  | NEX Glue vs. BioGlue | -0,7583 | **** | <0,0001 |
|  |  | Preveleak vs. BioGlue | -0,7777 | **** | <0,0001 |
|  | 260 | NEX Glue vs. Preveleak | 0,003333 | * | 0,0296 |
|  |  | NEX Glue vs. BioGlue | -0,9780 | **** | <0,0001 |
|  |  | Preveleak vs. BioGlue | -0,9813 | **** | <0,0001 |
|  | 270 | NEX Glue vs. Preveleak | -0,003333 | * | 0,0296 |
|  |  | NEX Glue vs. BioGlue | -1,242 | **** | <0,0001 |
|  |  | Preveleak vs. BioGlue | -1,238 | **** | <0,0001 |
|  | 280 | NEX Glue vs. Preveleak | -0,003000 | ** | 0,0094 |
|  |  | NEX Glue vs. BioGlue | -1,382 | **** | <0,0001 |
|  |  | Preveleak vs. BioGlue | -1,379 | **** | <0,0001 |
|  | 290 | NEX Glue vs. Preveleak | -0,001333 | ns | 0,1716 |
|  |  | NEX Glue vs. BioGlue | -1,187 | **** | <0,0001 |
|  |  | Preveleak vs. BioGlue | -1,186 | **** | <0,0001 |
|  | 300 | NEX Glue vs. Preveleak | 0,002333 | ns | 0,0594 |
|  |  | NEX Glue vs. BioGlue | -0,3753 | **** | <0,0001 |
|  |  | Preveleak vs. BioGlue | -0,3777 | **** | <0,0001 |
|  | 310 | NEX Glue vs. Preveleak | 0,004333 | * | 0,0176 |
|  |  | NEX Glue vs. BioGlue | -0,09767 | **** | <0,0001 |
|  |  | Preveleak vs. BioGlue | -0,1020 | **** | <0,0001 |
|  | 320 | NEX Glue vs. Preveleak | 0,005667 | *** | 0,0008 |
|  |  | NEX Glue vs. BioGlue | -0,04167 | *** | 0,0002 |
|  |  | Preveleak vs. BioGlue | -0,04733 | *** | 0,0001 |
|  | 330 | NEX Glue vs. Preveleak | 0,004333 | * | 0,0176 |
|  |  | NEX Glue vs. BioGlue | -0,02600 | **** | <0,0001 |
|  |  | Preveleak vs. BioGlue | -0,03033 | *** | 0,0004 |
|  | 340 | NEX Glue vs. Preveleak | 0,003333 | * | 0,0296 |
|  |  | NEX Glue vs. BioGlue | -0,02067 | *** | 0,0008 |
|  |  | Preveleak vs. BioGlue | -0,02400 | **** | <0,0001 |
|  | 350 | NEX Glue vs. Preveleak | 0,002000 | **** | <0,0001 |
|  |  | NEX Glue vs. BioGlue | -0,01600 | **** | <0,0001 |
|  |  | Preveleak vs. BioGlue | -0,01800 | **** | <0,0001 |
|  | 360 | NEX Glue vs. Preveleak | 0,0003333 | ns | >0,9999 |
|  |  | NEX Glue vs. BioGlue | -0,01300 | **** | <0,0001 |
|  |  | Preveleak vs. BioGlue | -0,01333 | ** | 0,0019 |


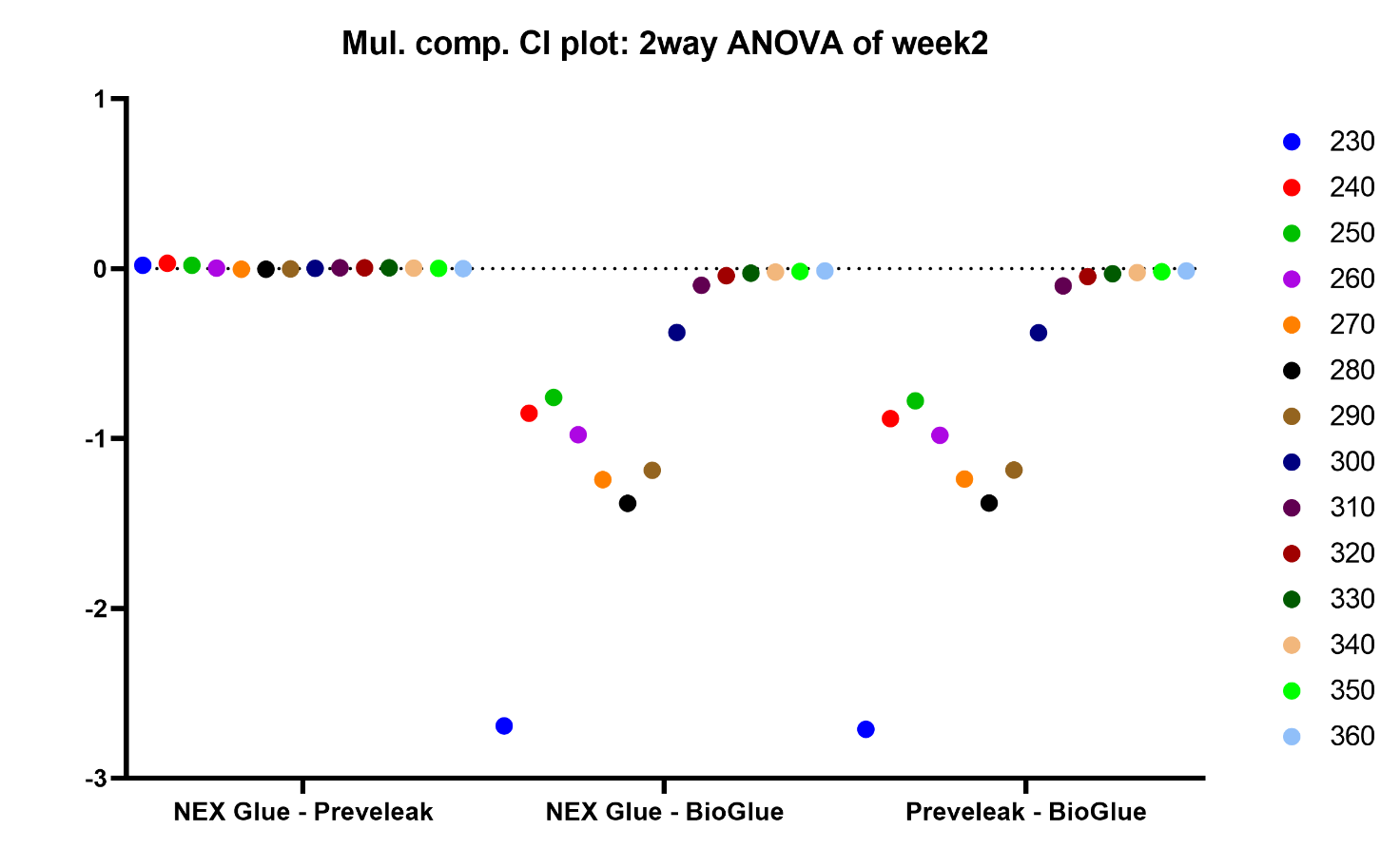


Table S13. Result of two-way ANOVA with Bonferroni's multiple comparisons test for degradation of surgical sealants for week 3

| **Week** | **Wavelength**  **[nm]** | **Bonferroni's multiple comparisons test** | **Mean Diff.** | **Summary** | **Adjusted P Value** |
| --- | --- | --- | --- | --- | --- |
| 3 | 230 | NEX Glue vs. Preveleak | 0,03133 | **** | <0,0001 |
|  |  | NEX Glue vs. BioGlue | -1,613 | *** | 0,0001 |
|  |  | Preveleak vs. BioGlue | -1,644 | *** | 0,0001 |
|  | 240 | NEX Glue vs. Preveleak | 0,03233 | **** | <0,0001 |
|  |  | NEX Glue vs. BioGlue | -0,4310 | **** | <0,0001 |
|  |  | Preveleak vs. BioGlue | -0,4633 | **** | <0,0001 |
|  | 250 | NEX Glue vs. Preveleak | 0,02067 | *** | 0,0001 |
|  |  | NEX Glue vs. BioGlue | -0,3843 | **** | <0,0001 |
|  |  | Preveleak vs. BioGlue | -0,4050 | **** | <0,0001 |
|  | 260 | NEX Glue vs. Preveleak | 0,007000 | ** | 0,0083 |
|  |  | NEX Glue vs. BioGlue | -0,4880 | **** | <0,0001 |
|  |  | Preveleak vs. BioGlue | -0,4950 | *** | 0,0001 |
|  | 270 | NEX Glue vs. Preveleak | 0,001667 | ns | 0,2473 |
|  |  | NEX Glue vs. BioGlue | -0,6103 | **** | <0,0001 |
|  |  | Preveleak vs. BioGlue | -0,6120 | *** | 0,0001 |
|  | 280 | NEX Glue vs. Preveleak | 0,003000 | ns | 0,0858 |
|  |  | NEX Glue vs. BioGlue | -0,6807 | **** | <0,0001 |
|  |  | Preveleak vs. BioGlue | -0,6837 | *** | 0,0001 |
|  | 290 | NEX Glue vs. Preveleak | 0,005333 | * | 0,0117 |
|  |  | NEX Glue vs. BioGlue | -0,5833 | *** | 0,0002 |
|  |  | Preveleak vs. BioGlue | -0,5887 | *** | 0,0002 |
|  | 300 | NEX Glue vs. Preveleak | 0,008333 | ** | 0,0024 |
|  |  | NEX Glue vs. BioGlue | -0,1847 | **** | <0,0001 |
|  |  | Preveleak vs. BioGlue | -0,1930 | **** | <0,0001 |
|  | 310 | NEX Glue vs. Preveleak | 0,01033 | **** | <0,0001 |
|  |  | NEX Glue vs. BioGlue | -0,04800 | **** | <0,0001 |
|  |  | Preveleak vs. BioGlue | -0,05833 | **** | <0,0001 |
|  | 320 | NEX Glue vs. Preveleak | 0,01100 | **** | <0,0001 |
|  |  | NEX Glue vs. BioGlue | -0,01800 | **** | <0,0001 |
|  |  | Preveleak vs. BioGlue | -0,02900 | **** | <0,0001 |
|  | 330 | NEX Glue vs. Preveleak | 0,01000 | **** | <0,0001 |
|  |  | NEX Glue vs. BioGlue | -0,01000 | **** | <0,0001 |
|  |  | Preveleak vs. BioGlue | -0,02000 | **** | <0,0001 |
|  | 340 | NEX Glue vs. Preveleak | 0,007000 | *** | 0,0004 |
|  |  | NEX Glue vs. BioGlue | -0,009000 | *** | 0,0001 |
|  |  | Preveleak vs. BioGlue | -0,01600 | **** | <0,0001 |
|  | 350 | NEX Glue vs. Preveleak | 0,004000 | ** | 0,0032 |
|  |  | NEX Glue vs. BioGlue | -0,009000 | *** | 0,0001 |
|  |  | Preveleak vs. BioGlue | -0,01300 | **** | <0,0001 |
|  | 360 | NEX Glue vs. Preveleak | 0,001333 | ns | 0,1716 |
|  |  | NEX Glue vs. BioGlue | -0,008667 | ** | 0,0044 |
|  |  | Preveleak vs. BioGlue | -0,01000 | **** | <0,0001 |


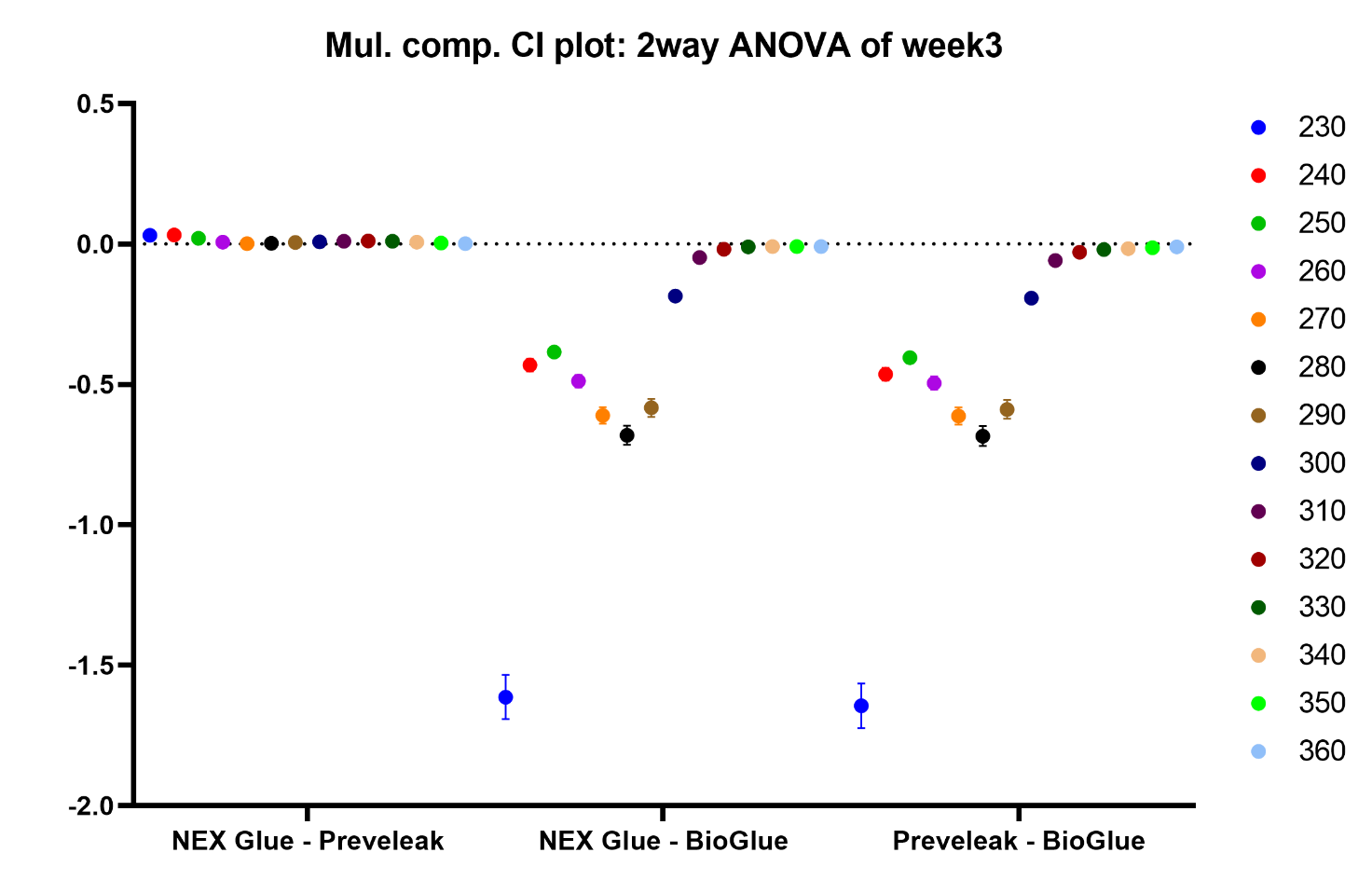


Table S14. Result of two-way ANOVA with Bonferroni's multiple comparisons test for degradation of surgical sealants for week 4

| **Week** | **Wavelength**  **[nm]** | **Bonferroni's multiple comparisons test** | **Mean Diff.** | **Summary** | **Adjusted P Value** |
| --- | --- | --- | --- | --- | --- |
| 4 | 230 | NEX Glue vs. Preveleak | 0,04767 | *** | 0,0004 |
|  |  | NEX Glue vs. BioGlue | -0,4943 | *** | 0,0010 |
|  |  | Preveleak vs. BioGlue | -0,5420 | *** | 0,0008 |
|  | 240 | NEX Glue vs. Preveleak | 0,03867 | *** | 0,0002 |
|  |  | NEX Glue vs. BioGlue | -0,1387 | *** | 0,0008 |
|  |  | Preveleak vs. BioGlue | -0,1773 | *** | 0,0005 |
|  | 250 | NEX Glue vs. Preveleak | 0,02700 | *** | 0,0006 |
|  |  | NEX Glue vs. BioGlue | -0,1210 | *** | 0,0005 |
|  |  | Preveleak vs. BioGlue | -0,1480 | *** | 0,0007 |
|  | 260 | NEX Glue vs. Preveleak | 0,01467 | ** | 0,0013 |
|  |  | NEX Glue vs. BioGlue | -0,1473 | *** | 0,0010 |
|  |  | Preveleak vs. BioGlue | -0,1620 | *** | 0,0009 |
|  | 270 | NEX Glue vs. Preveleak | 0,01000 | ** | 0,0013 |
|  |  | NEX Glue vs. BioGlue | -0,1773 | ** | 0,0011 |
|  |  | Preveleak vs. BioGlue | -0,1873 | ** | 0,0010 |
|  | 280 | NEX Glue vs. Preveleak | 0,01033 | ** | 0,0012 |
|  |  | NEX Glue vs. BioGlue | -0,1970 | ** | 0,0014 |
|  |  | Preveleak vs. BioGlue | -0,2073 | ** | 0,0011 |
|  | 290 | NEX Glue vs. Preveleak | 0,01100 | *** | 0,0009 |
|  |  | NEX Glue vs. BioGlue | -0,1710 | *** | 0,0010 |
|  |  | Preveleak vs. BioGlue | -0,1820 | *** | 0,0009 |
|  | 300 | NEX Glue vs. Preveleak | 0,01167 | **** | <0,0001 |
|  |  | NEX Glue vs. BioGlue | -0,05667 | ** | 0,0013 |
|  |  | Preveleak vs. BioGlue | -0,06833 | *** | 0,0009 |
|  | 310 | NEX Glue vs. Preveleak | 0,01267 | **** | <0,0001 |
|  |  | NEX Glue vs. BioGlue | -0,01400 | ** | 0,0045 |
|  |  | Preveleak vs. BioGlue | -0,02667 | *** | 0,0009 |
|  | 320 | NEX Glue vs. Preveleak | 0,01267 | ** | 0,0021 |
|  |  | NEX Glue vs. BioGlue | -0,002333 | ns | 0,1070 |
|  |  | Preveleak vs. BioGlue | -0,01500 | ** | 0,0044 |
|  | 330 | NEX Glue vs. Preveleak | 0,01067 | * | 0,0117 |
|  |  | NEX Glue vs. BioGlue | -0,0006667 | ns | >0,9999 |
|  |  | Preveleak vs. BioGlue | -0,01133 | ** | 0,0026 |
|  | 340 | NEX Glue vs. Preveleak | 0,008000 | *** | 0,0002 |
|  |  | NEX Glue vs. BioGlue | -0,001000 | ns | 0,3036 |
|  |  | Preveleak vs. BioGlue | -0,009000 | *** | 0,0001 |
|  | 350 | NEX Glue vs. Preveleak | 0,004667 | * | 0,0152 |
|  |  | NEX Glue vs. BioGlue | -0,003000 | ** | 0,0094 |
|  |  | Preveleak vs. BioGlue | -0,007667 | ** | 0,0057 |
|  | 360 | NEX Glue vs. Preveleak | 0,002000 | **** | <0,0001 |
|  |  | NEX Glue vs. BioGlue | -0,004000 | **** | <0,0001 |
|  |  | Preveleak vs. BioGlue | -0,006000 | **** | <0,0001 |


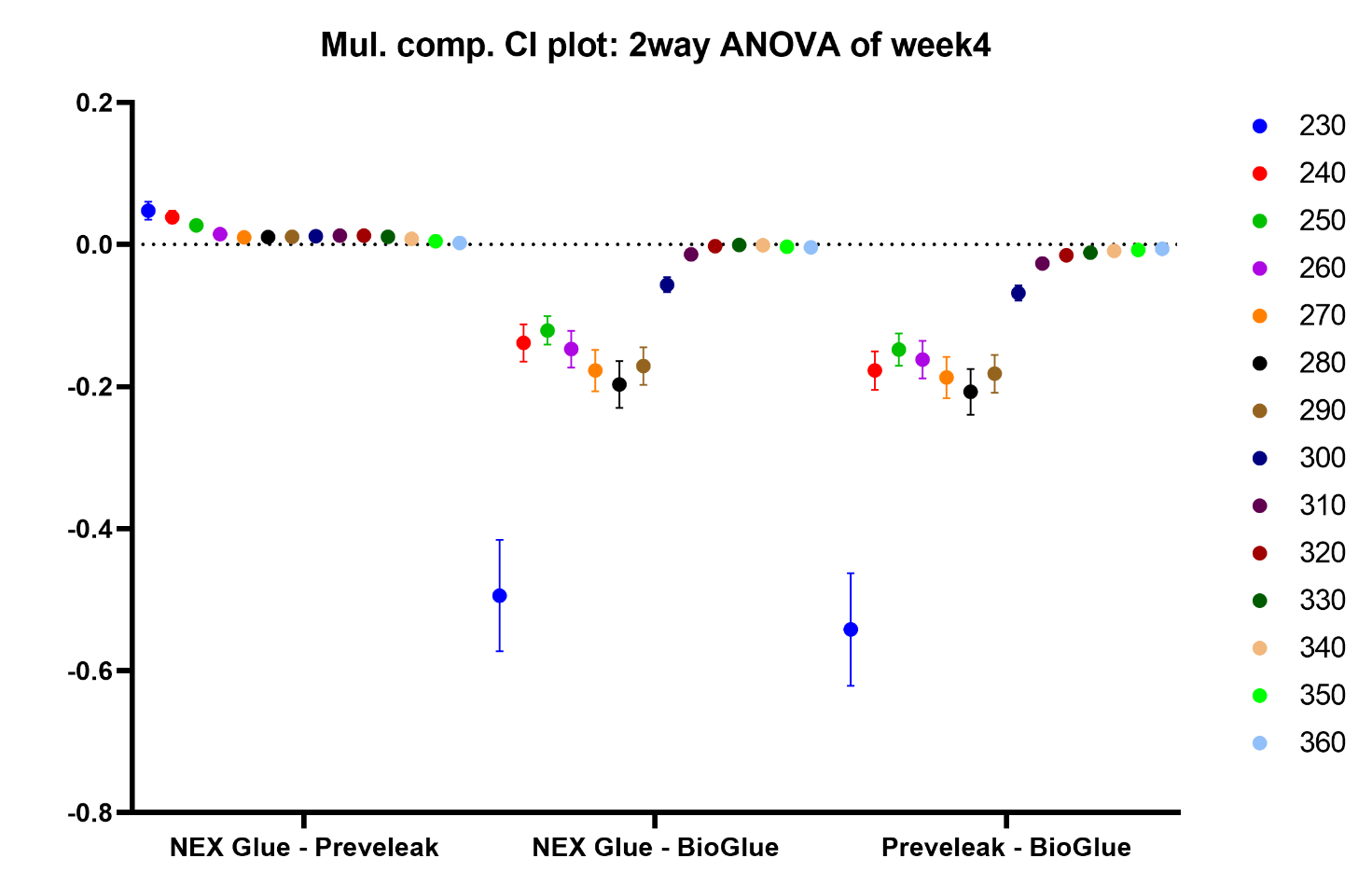


Table S15. Result of two-way ANOVA with Bonferroni's multiple comparisons test for degradation of surgical sealants for week 5

| **Week** | **Wavelength**  **[nm]** | **Bonferroni's multiple comparisons test** | **Mean Diff.** | **Summary** | **Adjusted P Value** |
| --- | --- | --- | --- | --- | --- |
| 5 | 230 | NEX Glue vs. Preveleak | 0,02633 | *** | 0,0002 |
|  |  | NEX Glue vs. BioGlue | -0,4253 | *** | 0,0001 |
|  |  | Preveleak vs. BioGlue | -0,4517 | *** | 0,0002 |
|  | 240 | NEX Glue vs. Preveleak | 0,02300 | **** | <0,0001 |
|  |  | NEX Glue vs. BioGlue | -0,1620 | *** | 0,0002 |
|  |  | Preveleak vs. BioGlue | -0,1850 | *** | 0,0001 |
|  | 250 | NEX Glue vs. Preveleak | 0,01433 | *** | 0,0004 |
|  |  | NEX Glue vs. BioGlue | -0,1310 | **** | <0,0001 |
|  |  | Preveleak vs. BioGlue | -0,1453 | *** | 0,0002 |
|  | 260 | NEX Glue vs. Preveleak | 0,005667 | *** | 0,0008 |
|  |  | NEX Glue vs. BioGlue | -0,1360 | **** | <0,0001 |
|  |  | Preveleak vs. BioGlue | -0,1417 | **** | <0,0001 |
|  | 270 | NEX Glue vs. Preveleak | 0,003333 | * | 0,0296 |
|  |  | NEX Glue vs. BioGlue | -0,1477 | *** | 0,0002 |
|  |  | Preveleak vs. BioGlue | -0,1510 | *** | 0,0003 |
|  | 280 | NEX Glue vs. Preveleak | 0,004000 | ** | 0,0032 |
|  |  | NEX Glue vs. BioGlue | -0,1600 | **** | <0,0001 |
|  |  | Preveleak vs. BioGlue | -0,1640 | **** | <0,0001 |
|  | 290 | NEX Glue vs. Preveleak | 0,005000 | ** | 0,0013 |
|  |  | NEX Glue vs. BioGlue | -0,1427 | **** | <0,0001 |
|  |  | Preveleak vs. BioGlue | -0,1477 | **** | <0,0001 |
|  | 300 | NEX Glue vs. Preveleak | 0,006333 | ** | 0,0083 |
|  |  | NEX Glue vs. BioGlue | -0,06100 | **** | <0,0001 |
|  |  | Preveleak vs. BioGlue | -0,06733 | *** | 0,0003 |
|  | 310 | NEX Glue vs. Preveleak | 0,007333 | ** | 0,0036 |
|  |  | NEX Glue vs. BioGlue | -0,02567 | **** | <0,0001 |
|  |  | Preveleak vs. BioGlue | -0,03300 | **** | <0,0001 |
|  | 320 | NEX Glue vs. Preveleak | 0,007333 | ** | 0,0062 |
|  |  | NEX Glue vs. BioGlue | -0,01367 | ** | 0,0018 |
|  |  | Preveleak vs. BioGlue | -0,02100 | **** | <0,0001 |
|  | 330 | NEX Glue vs. Preveleak | 0,006667 | *** | 0,0004 |
|  |  | NEX Glue vs. BioGlue | -0,008667 | ** | 0,0044 |
|  |  | Preveleak vs. BioGlue | -0,01533 | ** | 0,0014 |
|  | 340 | NEX Glue vs. Preveleak | 0,004667 | * | 0,0152 |
|  |  | NEX Glue vs. BioGlue | -0,007667 | *** | 0,0003 |
|  |  | Preveleak vs. BioGlue | -0,01233 | ** | 0,0022 |
|  | 350 | NEX Glue vs. Preveleak | 0,003667 | ** | 0,0044 |
|  |  | NEX Glue vs. BioGlue | -0,007000 | *** | 0,0004 |
|  |  | Preveleak vs. BioGlue | -0,01067 | **** | <0,0001 |
|  | 360 | NEX Glue vs. Preveleak | 0,001000 | ns | 0,3036 |
|  |  | NEX Glue vs. BioGlue | -0,007667 | ** | 0,0057 |
|  |  | Preveleak vs. BioGlue | -0,008667 | ** | 0,0044 |


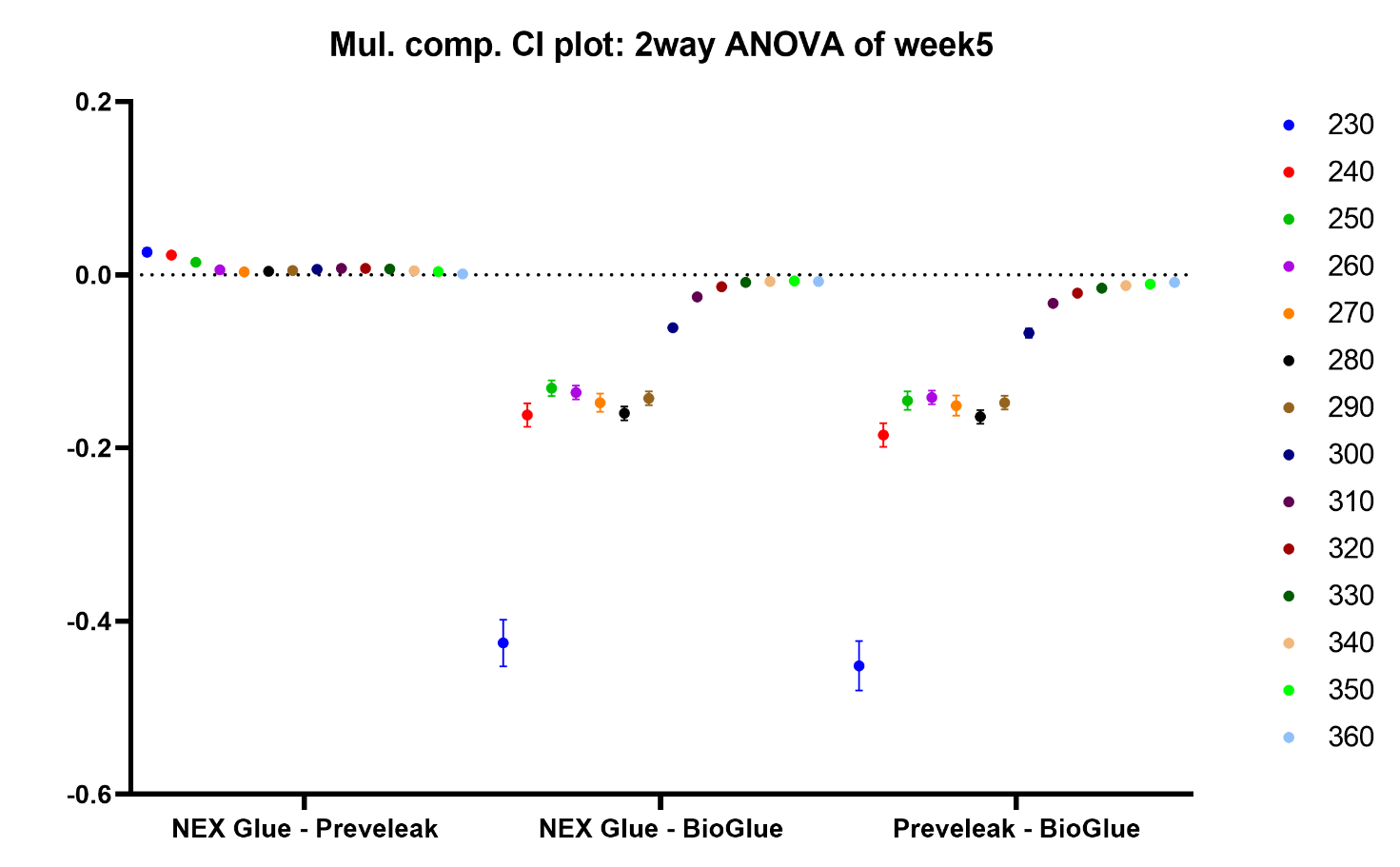


Table S16. Result of two-way ANOVA with Bonferroni's multiple comparisons test for degradation of surgical sealants for week 6

| **Week** | **Wavelength**  **[nm]** | **Bonferroni's multiple comparisons test** | **Mean Diff.** | **Summary** | **Adjusted P Value** |
| --- | --- | --- | --- | --- | --- |
| 6 | 230 | NEX Glue vs. Preveleak | -0,009333 | ns | >0,9999 |
|  |  | NEX Glue vs. BioGlue | -0,2037 | **** | <0,0001 |
|  |  | Preveleak vs. BioGlue | -0,1943 | * | 0,0436 |
|  | 240 | NEX Glue vs. Preveleak | 0,01100 | ns | 0,1042 |
|  |  | NEX Glue vs. BioGlue | -0,09200 | **** | <0,0001 |
|  |  | Preveleak vs. BioGlue | -0,1030 | *** | 0,0005 |
|  | 250 | NEX Glue vs. Preveleak | 0,006667 | ns | 0,1297 |
|  |  | NEX Glue vs. BioGlue | -0,07200 | **** | <0,0001 |
|  |  | Preveleak vs. BioGlue | -0,07867 | *** | 0,0001 |
|  | 260 | NEX Glue vs. Preveleak | -0,0003333 | ns | >0,9999 |
|  |  | NEX Glue vs. BioGlue | -0,06867 | **** | <0,0001 |
|  |  | Preveleak vs. BioGlue | -0,06833 | ** | 0,0034 |
|  | 270 | NEX Glue vs. Preveleak | -0,003667 | ns | >0,9999 |
|  |  | NEX Glue vs. BioGlue | -0,06967 | **** | <0,0001 |
|  |  | Preveleak vs. BioGlue | -0,06600 | * | 0,0123 |
|  | 280 | NEX Glue vs. Preveleak | -0,003333 | ns | >0,9999 |
|  |  | NEX Glue vs. BioGlue | -0,07367 | **** | <0,0001 |
|  |  | Preveleak vs. BioGlue | -0,07033 | * | 0,0118 |
|  | 290 | NEX Glue vs. Preveleak | 0,0006667 | ns | >0,9999 |
|  |  | NEX Glue vs. BioGlue | -0,06767 | **** | <0,0001 |
|  |  | Preveleak vs. BioGlue | -0,06833 | *** | 0,0005 |
|  | 300 | NEX Glue vs. Preveleak | 0,002000 | ns | 0,2294 |
|  |  | NEX Glue vs. BioGlue | -0,03333 | *** | 0,0003 |
|  |  | Preveleak vs. BioGlue | -0,03533 | ** | 0,0011 |
|  | 310 | NEX Glue vs. Preveleak | 0,003333 | ns | 0,1132 |
|  |  | NEX Glue vs. BioGlue | -0,01700 | **** | <0,0001 |
|  |  | Preveleak vs. BioGlue | -0,02033 | ** | 0,0032 |
|  | 320 | NEX Glue vs. Preveleak | 0,003667 | ns | 0,1652 |
|  |  | NEX Glue vs. BioGlue | -0,01033 | ** | 0,0031 |
|  |  | Preveleak vs. BioGlue | -0,01400 | * | 0,0152 |
|  | 330 | NEX Glue vs. Preveleak | 0,003000 | ns | 0,3636 |
|  |  | NEX Glue vs. BioGlue | -0,006333 | ** | 0,0083 |
|  |  | Preveleak vs. BioGlue | -0,009333 | * | 0,0485 |
|  | 340 | NEX Glue vs. Preveleak | 0,001667 | ns | 0,6005 |
|  |  | NEX Glue vs. BioGlue | -0,006000 | * | 0,0274 |
|  |  | Preveleak vs. BioGlue | -0,007667 | * | 0,0389 |
|  | 350 | NEX Glue vs. Preveleak | 0,001333 | ns | 0,9398 |
|  |  | NEX Glue vs. BioGlue | -0,005333 | ** | 0,0010 |
|  |  | Preveleak vs. BioGlue | -0,006667 | * | 0,0433 |
|  | 360 | NEX Glue vs. Preveleak | 0,0003333 | ns | >0,9999 |
|  |  | NEX Glue vs. BioGlue | -0,005333 | ** | 0,0096 |
|  |  | Preveleak vs. BioGlue | -0,005667 | * | 0,0152 |


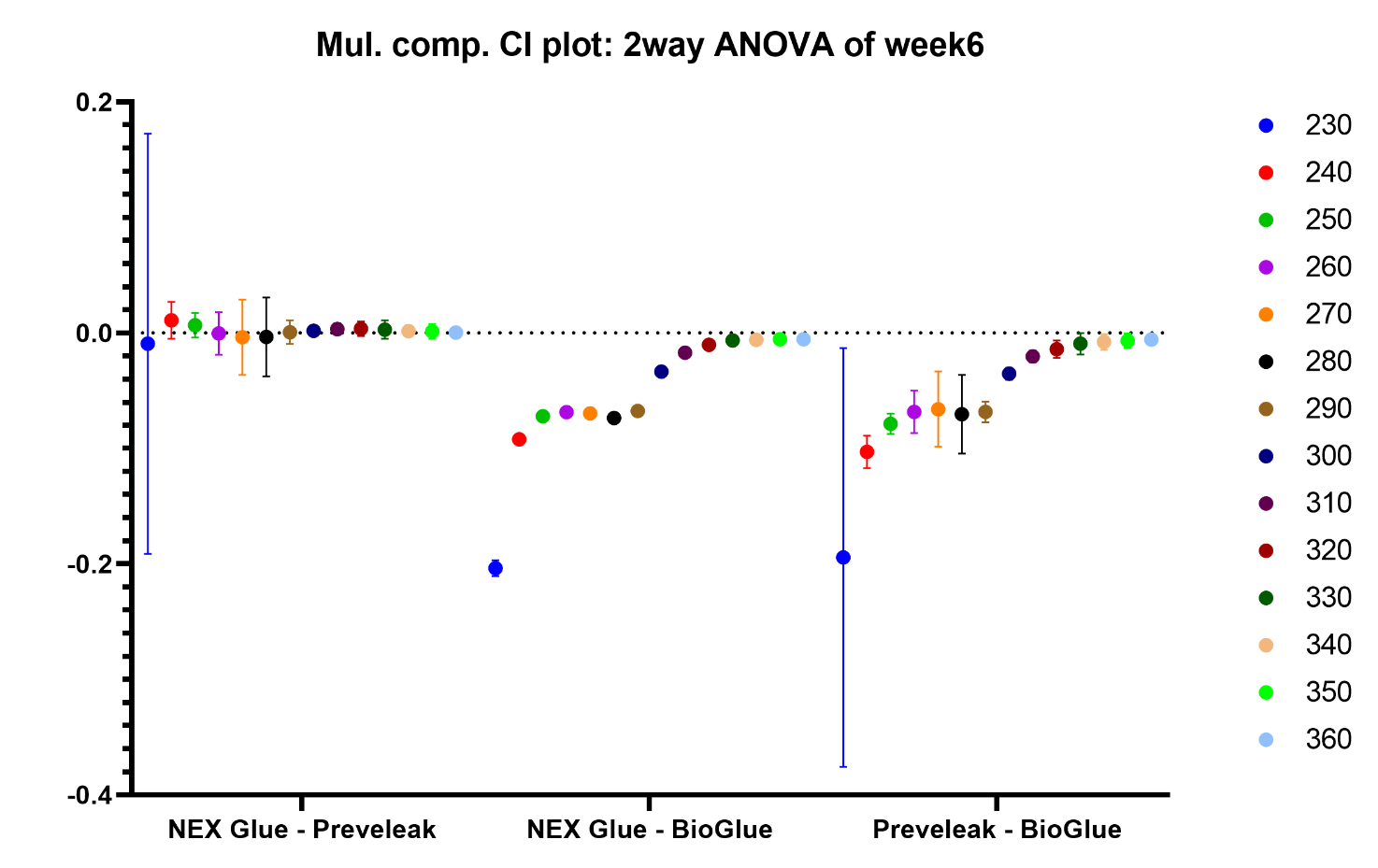


Table S17. Result of two-way ANOVA with Bonferroni's multiple comparisons test for degradation of surgical sealants for week 7

| **Week** | **Wavelength**  **[nm]** | **Bonferroni's multiple comparisons test** | **Mean Diff.** | **Summary** | **Adjusted P Value** |
| --- | --- | --- | --- | --- | --- |
| 7 | 230 | NEX Glue vs. Preveleak | 0,006000 | ns | 0,8079 |
|  |  | NEX Glue vs. BioGlue | -0,1170 | **** | <0,0001 |
|  |  | Preveleak vs. BioGlue | -0,1230 | *** | 0,0002 |
|  | 240 | NEX Glue vs. Preveleak | 0,004667 | ns | >0,9999 |
|  |  | NEX Glue vs. BioGlue | -0,06267 | *** | 0,0002 |
|  |  | Preveleak vs. BioGlue | -0,06733 | ** | 0,0059 |
|  | 250 | NEX Glue vs. Preveleak | 0,001333 | ns | >0,9999 |
|  |  | NEX Glue vs. BioGlue | -0,04700 | *** | 0,0002 |
|  |  | Preveleak vs. BioGlue | -0,04833 | * | 0,0196 |
|  | 260 | NEX Glue vs. Preveleak | -0,002333 | ns | >0,9999 |
|  |  | NEX Glue vs. BioGlue | -0,04200 | *** | 0,0002 |
|  |  | Preveleak vs. BioGlue | -0,03967 | * | 0,0246 |
|  | 270 | NEX Glue vs. Preveleak | -0,004667 | ns | >0,9999 |
|  |  | NEX Glue vs. BioGlue | -0,04033 | **** | <0,0001 |
|  |  | Preveleak vs. BioGlue | -0,03567 | * | 0,0397 |
|  | 280 | NEX Glue vs. Preveleak | -0,004000 | ns | >0,9999 |
|  |  | NEX Glue vs. BioGlue | -0,04100 | *** | 0,0003 |
|  |  | Preveleak vs. BioGlue | -0,03700 | * | 0,0329 |
|  | 290 | NEX Glue vs. Preveleak | -0,002667 | ns | >0,9999 |
|  |  | NEX Glue vs. BioGlue | -0,03767 | *** | 0,0004 |
|  |  | Preveleak vs. BioGlue | -0,03500 | * | 0,0370 |
|  | 300 | NEX Glue vs. Preveleak | -0,002000 | ns | >0,9999 |
|  |  | NEX Glue vs. BioGlue | -0,02300 | **** | <0,0001 |
|  |  | Preveleak vs. BioGlue | -0,02100 | ns | 0,0966 |
|  | 310 | NEX Glue vs. Preveleak | -0,002000 | ns | >0,9999 |
|  |  | NEX Glue vs. BioGlue | -0,01333 | *** | 0,0005 |
|  |  | Preveleak vs. BioGlue | -0,01133 | ns | 0,3128 |
|  | 320 | NEX Glue vs. Preveleak | -0,0006667 | ns | >0,9999 |
|  |  | NEX Glue vs. BioGlue | -0,008000 | *** | 0,0002 |
|  |  | Preveleak vs. BioGlue | -0,007333 | ns | 0,6225 |
|  | 330 | NEX Glue vs. Preveleak | -0,0006667 | ns | >0,9999 |
|  |  | NEX Glue vs. BioGlue | -0,005667 | ** | 0,0080 |
|  |  | Preveleak vs. BioGlue | -0,005000 | ns | >0,9999 |
|  | 340 | NEX Glue vs. Preveleak | -0,001333 | ns | >0,9999 |
|  |  | NEX Glue vs. BioGlue | -0,005000 | ** | 0,0013 |
|  |  | Preveleak vs. BioGlue | -0,003667 | ns | >0,9999 |
|  | 350 | NEX Glue vs. Preveleak | -0,002667 | ns | >0,9999 |
|  |  | NEX Glue vs. BioGlue | -0,005000 | **** | <0,0001 |
|  |  | Preveleak vs. BioGlue | -0,002333 | ns | >0,9999 |
|  | 360 | NEX Glue vs. Preveleak | -0,003333 | ns | >0,9999 |
|  |  | NEX Glue vs. BioGlue | -0,004333 | ** | 0,0023 |
|  |  | Preveleak vs. BioGlue | -0,001000 | ns | >0,9999 |


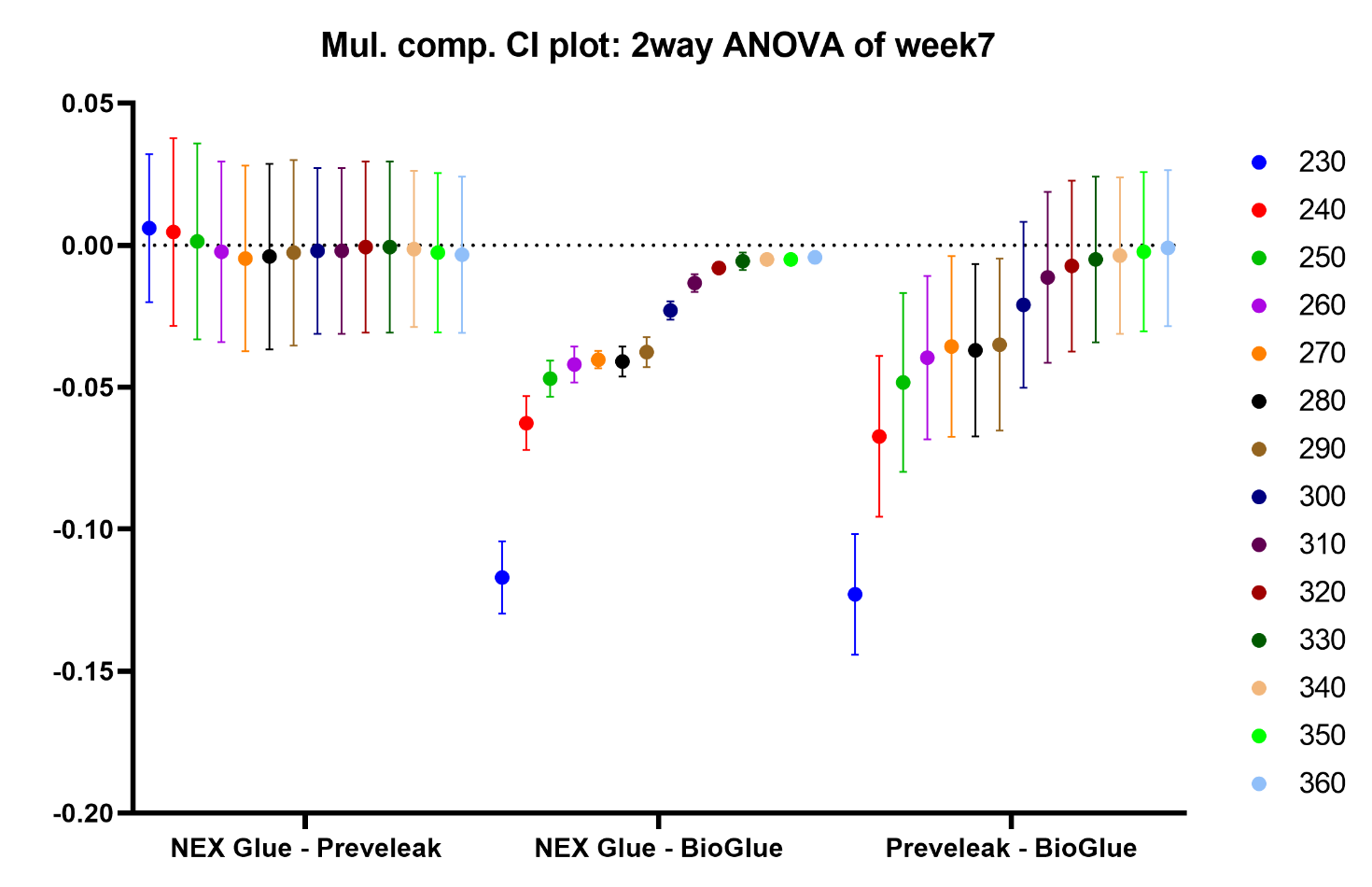


Table S18. Result of two-way ANOVA with Bonferroni's multiple comparisons test for degradation of surgical sealants for week 8

| **Week** | **Wavelength**  **[nm]** | **Bonferroni's multiple comparisons test** | **Mean Diff.** | **Summary** | **Adjusted P Value** |
| --- | --- | --- | --- | --- | --- |
| 8 | 230 | NEX Glue vs. Preveleak | 0,01667 | * | 0,0165 |
|  |  | NEX Glue vs. BioGlue | -0,06700 | **** | <0,0001 |
|  |  | Preveleak vs. BioGlue | -0,08367 | *** | 0,0002 |
|  | 240 | NEX Glue vs. Preveleak | 0,01433 | * | 0,0261 |
|  |  | NEX Glue vs. BioGlue | -0,04167 | *** | 0,0003 |
|  |  | Preveleak vs. BioGlue | -0,05600 | *** | 0,0001 |
|  | 250 | NEX Glue vs. Preveleak | 0,009667 | ** | 0,0068 |
|  |  | NEX Glue vs. BioGlue | -0,03033 | **** | <0,0001 |
|  |  | Preveleak vs. BioGlue | -0,04000 | **** | <0,0001 |
|  | 260 | NEX Glue vs. Preveleak | 0,004667 | ns | 0,1265 |
|  |  | NEX Glue vs. BioGlue | -0,02633 | ** | 0,0011 |
|  |  | Preveleak vs. BioGlue | -0,03100 | **** | <0,0001 |
|  | 270 | NEX Glue vs. Preveleak | 0,002333 | ns | 0,4022 |
|  |  | NEX Glue vs. BioGlue | -0,02433 | *** | 0,0002 |
|  |  | Preveleak vs. BioGlue | -0,02667 | *** | 0,0009 |
|  | 280 | NEX Glue vs. Preveleak | 0,003000 | ns | 0,1729 |
|  |  | NEX Glue vs. BioGlue | -0,02367 | *** | 0,0002 |
|  |  | Preveleak vs. BioGlue | -0,02667 | **** | <0,0001 |
|  | 290 | NEX Glue vs. Preveleak | 0,003333 | ns | 0,0723 |
|  |  | NEX Glue vs. BioGlue | -0,02200 | **** | <0,0001 |
|  |  | Preveleak vs. BioGlue | -0,02533 | **** | <0,0001 |
|  | 300 | NEX Glue vs. Preveleak | 0,004333 | ns | 0,1151 |
|  |  | NEX Glue vs. BioGlue | -0,01467 | ** | 0,0017 |
|  |  | Preveleak vs. BioGlue | -0,01900 | *** | 0,0005 |
|  | 310 | NEX Glue vs. Preveleak | 0,004667 | ns | 0,0683 |
|  |  | NEX Glue vs. BioGlue | -0,009333 | ** | 0,0044 |
|  |  | Preveleak vs. BioGlue | -0,01400 | ** | 0,0011 |
|  | 320 | NEX Glue vs. Preveleak | 0,005000 | * | 0,0216 |
|  |  | NEX Glue vs. BioGlue | -0,006000 | ** | 0,0094 |
|  |  | Preveleak vs. BioGlue | -0,01100 | ** | 0,0023 |
|  | 330 | NEX Glue vs. Preveleak | 0,004333 | ns | 0,1151 |
|  |  | NEX Glue vs. BioGlue | -0,005000 | ns | 0,0597 |
|  |  | Preveleak vs. BioGlue | -0,009333 | ** | 0,0017 |
|  | 340 | NEX Glue vs. Preveleak | 0,003333 | * | 0,0395 |
|  |  | NEX Glue vs. BioGlue | -0,003667 | * | 0,0299 |
|  |  | Preveleak vs. BioGlue | -0,007000 | ** | 0,0030 |
|  | 350 | NEX Glue vs. Preveleak | 0,002333 | ns | 0,1070 |
|  |  | NEX Glue vs. BioGlue | -0,003667 | * | 0,0443 |
|  |  | Preveleak vs. BioGlue | -0,006000 | * | 0,0129 |
|  | 360 | NEX Glue vs. Preveleak | 0,001667 | ns | 0,3888 |
|  |  | NEX Glue vs. BioGlue | -0,003000 | ns | 0,1004 |
|  |  | Preveleak vs. BioGlue | -0,004667 | ns | 0,0594 |

**
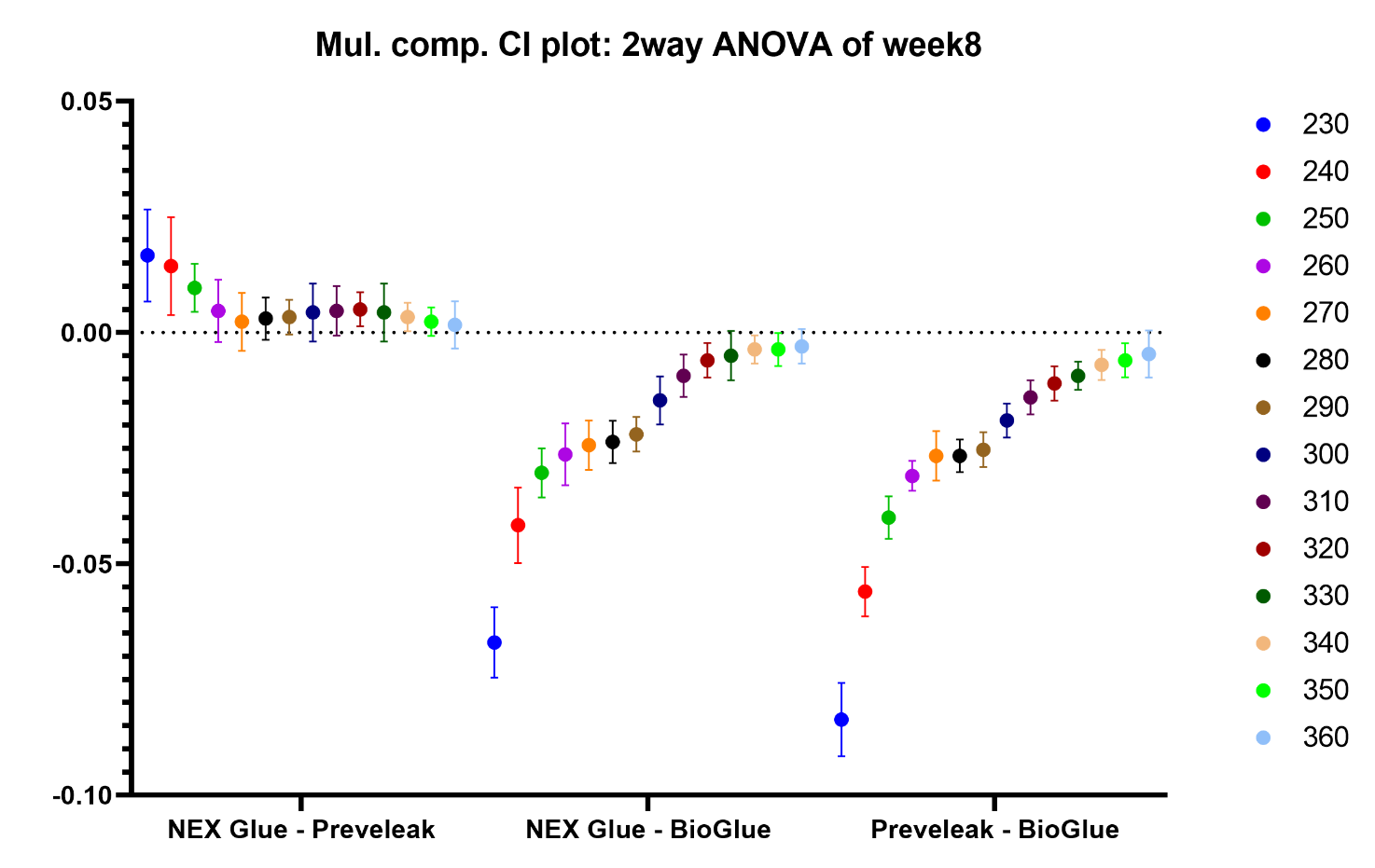
**

Table S19. Result of two-way ANOVA with Bonferroni's multiple comparisons test for degradation of surgical sealants for week 9

| **Week** | **Wavelength**  **[nm]** | **Bonferroni's multiple comparisons test** | **Mean Diff.** | **Summary** | **Adjusted P Value** |
| --- | --- | --- | --- | --- | --- |
| 9 | 230 | NEX Glue vs. Preveleak | 0,007000 | ns | 0,0957 |
|  |  | NEX Glue vs. BioGlue | -0,03333 | * | 0,0418 |
|  |  | Preveleak vs. BioGlue | -0,04033 | * | 0,0395 |
|  | 240 | NEX Glue vs. Preveleak | 0,005667 | ns | 0,0993 |
|  |  | NEX Glue vs. BioGlue | -0,02433 | ns | 0,0821 |
|  |  | Preveleak vs. BioGlue | -0,03000 | ns | 0,0646 |
|  | 250 | NEX Glue vs. Preveleak | 0,004333 | ns | 0,1151 |
|  |  | NEX Glue vs. BioGlue | -0,01733 | ns | 0,1525 |
|  |  | Preveleak vs. BioGlue | -0,02167 | ns | 0,1112 |
|  | 260 | NEX Glue vs. Preveleak | 0,001667 | ns | 0,6965 |
|  |  | NEX Glue vs. BioGlue | -0,01567 | ns | 0,1720 |
|  |  | Preveleak vs. BioGlue | -0,01733 | ns | 0,1578 |
|  | 270 | NEX Glue vs. Preveleak | 0,001333 | ns | >0,9999 |
|  |  | NEX Glue vs. BioGlue | -0,01433 | ns | 0,1709 |
|  |  | Preveleak vs. BioGlue | -0,01567 | ns | 0,1545 |
|  | 280 | NEX Glue vs. Preveleak | 0,001000 | ns | >0,9999 |
|  |  | NEX Glue vs. BioGlue | -0,01367 | ns | 0,1690 |
|  |  | Preveleak vs. BioGlue | -0,01467 | ns | 0,1620 |
|  | 290 | NEX Glue vs. Preveleak | 0,001667 | ns | 0,9100 |
|  |  | NEX Glue vs. BioGlue | -0,01267 | ns | 0,2098 |
|  |  | Preveleak vs. BioGlue | -0,01433 | ns | 0,1860 |
|  | 300 | NEX Glue vs. Preveleak | 0,001667 | ns | 0,9100 |
|  |  | NEX Glue vs. BioGlue | -0,01033 | ns | 0,2581 |
|  |  | Preveleak vs. BioGlue | -0,01200 | ns | 0,2170 |
|  | 310 | NEX Glue vs. Preveleak | 0,002000 | ns | 0,4183 |
|  |  | NEX Glue vs. BioGlue | -0,008333 | ns | 0,4334 |
|  |  | Preveleak vs. BioGlue | -0,01033 | ns | 0,3151 |
|  | 320 | NEX Glue vs. Preveleak | 0,002000 | ns | 0,7144 |
|  |  | NEX Glue vs. BioGlue | -0,006667 | ns | 0,5223 |
|  |  | Preveleak vs. BioGlue | -0,008667 | ns | 0,3491 |
|  | 330 | NEX Glue vs. Preveleak | 0,002000 | ns | 0,3036 |
|  |  | NEX Glue vs. BioGlue | -0,006000 | ns | 0,5872 |
|  |  | Preveleak vs. BioGlue | -0,008000 | ns | 0,3683 |
|  | 340 | NEX Glue vs. Preveleak | 0,001667 | ns | 0,6339 |
|  |  | NEX Glue vs. BioGlue | -0,005000 | ns | 0,6458 |
|  |  | Preveleak vs. BioGlue | -0,006667 | ns | 0,4132 |
|  | 350 | NEX Glue vs. Preveleak | 0,001000 | ns | >0,9999 |
|  |  | NEX Glue vs. BioGlue | -0,004667 | ns | 0,7690 |
|  |  | Preveleak vs. BioGlue | -0,005667 | ns | 0,5946 |
|  | 360 | NEX Glue vs. Preveleak | 0,001000 | ns | >0,9999 |
|  |  | NEX Glue vs. BioGlue | -0,004333 | ns | 0,7225 |
|  |  | Preveleak vs. BioGlue | -0,005333 | ns | 0,5452 |

**
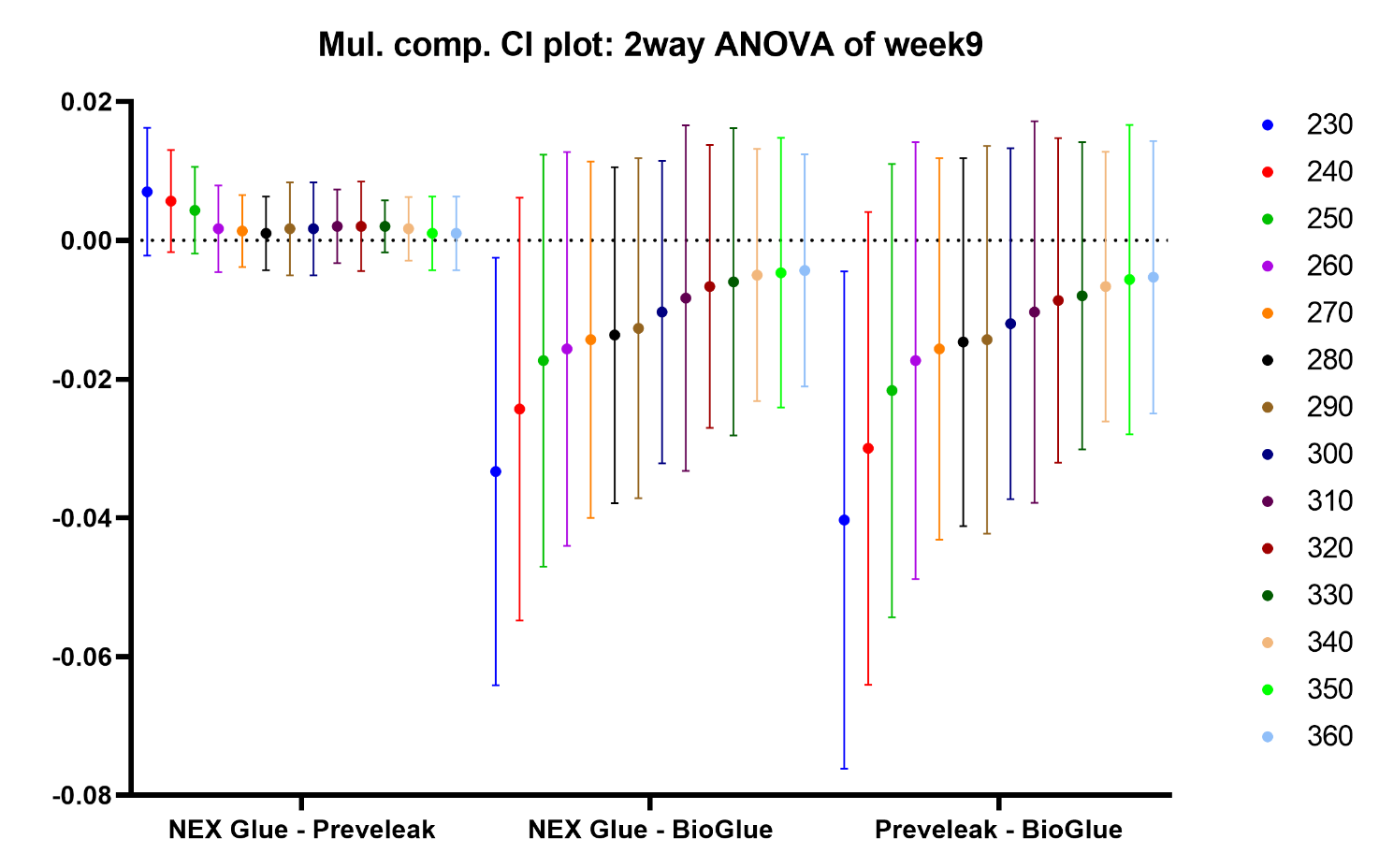
**
